# An Unbiased Functional Genetics Screen Identifies Rare Activating ERBB4 Mutations

**DOI:** 10.1158/2767-9764.CRC-21-0021

**Published:** 2022-01-07

**Authors:** Deepankar Chakroborty, Veera K. Ojala, Anna M. Knittle, Jasmin Drexler, Mahlet Z. Tamirat, Regina Ruzicka, Karin Bosch, Johanna Woertl, Susanne Schmittner, Laura L. Elo, Mark S. Johnson, Kari J. Kurppa, Flavio Solca, Klaus Elenius

**Affiliations:** 1 Institute of Biomedicine, University of Turku, Turku, Finland.; 2 Medicity Research Laboratories, University of Turku, Turku, Finland.; 3 Turku Doctoral Programme of Molecular Medicine, Turku, Finland.; 4 Turku Bioscience Centre, University of Turku and Åbo Akademi University, Turku, Finland.; 5 Boehringer Ingelheim RCV GmbH & Co KG, Vienna, Austria.; 6 Structural Bioinformatics Laboratory, Biochemistry, Faculty of Science and Engineering, Åbo Akademi University, Turku, Finland.; 7 InFLAMES Research Flagship Center, Åbo Akademi University, Turku, Finland.; 8 Graduate School of Åbo Akademi University (Informational and Structural Biology Doctoral Network), Turku, Finland.; 9 Department of Oncology, Turku University Hospital, Turku, Finland.

## Abstract

Despite the relatively high frequency of somatic *ERBB4* mutations in various cancer types, only a few activating *ERBB4* mutations have been characterized, primarily due to lack of mutational hotspots in the *ERBB4* gene. Here, we utilized our previously published pipeline, an *in vitro* screen for activating mutations, to perform an unbiased functional screen to identify potential activating ERBB4 mutations from a randomly mutated *ERBB4* expression library. Ten potentially activating *ERBB4* mutations were identified and subjected to validation by functional and structural analyses. Two of the 10 ERBB4 mutants, E715K and R687K, demonstrated hyperactivity in all tested cell models and promoted cellular growth under two-dimensional and three-dimensional culture conditions. ERBB4 E715K also promoted tumor growth in *in vivo* Ba/F3 cell mouse allografts. Importantly, all tested ERBB4 mutants were sensitive to the pan-ERBB tyrosine kinase inhibitors afatinib, neratinib, and dacomitinib. Our data indicate that rare *ERBB4* mutations are potential candidates for ERBB4-targeted therapy with pan-ERBB inhibitors.

**Statement of Significance:**

ERBB4 is a member of the ERBB family of oncogenes that is frequently mutated in different cancer types but the functional impact of its somatic mutations remains unknown. Here, we have analyzed the function of over 8,000 randomly mutated ERBB4 variants in an unbiased functional genetics screen. The data indicate the presence of rare activating ERBB4 mutations in cancer, with potential to be targeted with clinically approved pan-ERBB inhibitors.

## Introduction

The ERBB family of proteins contains four structurally homologous receptor tyrosine kinases (RTK): EGFR or (ERBB1), ERBB2, ERBB3, and ERBB4. The ERBB receptors are proto-oncogenes and several cancer drugs targeting ERBB receptors have been introduced into clinical practice (1, 2). Of the four family members, EGFR and ERBB4 are canonical RTKs with a ligand-binding extracellular domain and a fully functional intracellular tyrosine kinase domain (3, 4). ERBB2 and ERBB3, on the other hand, have impaired ligand-binding and intrinsic tyrosine kinase activity, respectively (5, 6).

ERBB4 is a 180 kDa glycoprotein that is unique among the ERBB family members in that it undergoes alternative splicing to produce four different naturally occurring variants from a single allele (7, 8). The juxtamembrane (JM) isoforms JM-a and JM-b differ within their extracellular JM domains with the JM-a–specific sequence providing a proteolytic cleavage site that is missing from JM-b (7). The cytoplasmic (CYT) isoforms differ in their cytoplasmic domains by including an amino acid sequence that can provide the receptor a direct binding site for PI3-K or WW domain-containing ubiquitin ligases in the case of CYT-1 isoform (9, 10). This CYT-1–specific sequence is missing from the CYT-2 isoform of ERBB4 (8).

ERBB4 is expressed, for example, in neural tissues, kidney, skeletal muscle, and the heart (11–13). ERBB4 is also expressed in several cancer cell lines (Supplementary Fig. S1A) and is found frequently mutated in various cancer types (Supplementary Fig. S1B), with frequencies ranging from over 15% in melanoma to less than 1% in leukemia (https://genie.cbioportal.org). Functional annotation has been reported for a few ERBB4 mutations with evidence for both gain of function (14) as well as loss of function (15). However, for the great majority of the hundreds of reported somatic ERBB4 mutations, there is no functional information available. Such molecular information would facilitate understanding the role these mutations can play as potential predictive markers for targeted therapy.

The ERBB-targeting drugs approved for clinical use include the first-generation tyrosine kinase inhibitors (TKI) and mAbs blocking the signaling of either EGFR and/or ERBB2. More recently, irreversible second-generation TKIs afatinib, neratinib, and dacomitinib have been approved for treatment of *EGFR*-mutant non–small cell lung cancer and *ERBB2* amplification-positive breast cancer (www.fda.gov). Collectively, these second-generation ERBB TKIs are called pan-ERBB inhibitors, as they, in addition to EGFR and ERBB2, also inhibit the kinase activity of ERBB4 (16). Specifically, afatinib has been shown to covalently irreversibly bind ERBB4 and inhibit its kinase activity (17).

Despite a relatively high mutational burden in cancer tissues (18, 19), only a few predictive genetic markers are currently used to direct treatment decisions in the clinic (20). Identification of driver mutations, and, consequently, of predictive markers is challenging as most of the cancer-associated somatic mutations are expected to be inconsequential passengers (21). Moreover, a significant proportion of functional drivers are present at a low frequency in the mutational landscape (22). Here we used an unbiased functional genetics assay to systematically screen thousands of randomly mutated *ERBB4* variants to identify gain-of-function mutations. Our findings indicate that *ERBB4* harbors functionally active somatic mutations and that these mutations are rare in clinical databases. Of the individual ERBB4 variants we demonstrate that R687K and E715K are active in *in vitro* and *in vivo* models, and present mechanistic insights behind their activity.

## Materials and Methods

### Plasmids

For transient overexpression of ERBB receptors, previously described constructs *pcDNA3.1ERBB3-HA* (23), *pcDNA3.1ERBB4JM-aCYT-2-K751R-HA* (15), and *pcDNA3.1ERBB4JM-aCYT-2-V956R-HA* (14) were used.

For stable overexpression of ERBB receptors, wild-type human *ERBB4* cDNA encoding JM-a CYT-1 or JM-a CYT-2 were amplified respectively from *pcDNA3.1neo(−)-ERBB4JM-aCYT-1* (24) or *pcDNA3.1neo(−)-ERBB4JM-aCYT-2* (24) using the primer pair 5′-ggggacaagtttgtacaaaaaagcaggcttcaccatgcgaccggctacaggact-3′ and 5′-ggggaccactttgtacaagaaagctgggttttacaccacagtattccggtgtc-3′ to generate amplicons flanked by attB1 and attB2 sites. The amplicons were cloned into the *pDONR221* vector (Tol2Kit; ref. 25) using BP clonase II mix (Invitrogen) to generate *pDONR221-ERBB4JM-aCYT-1* and *pDONR221-ERBB4JM-aCYT-2*. Retroviral mammalian expression constructs were made with a LR-Gateway recombination reaction of the *pDONR221*-based plasmids with *pBABEpuro-gateway* (26), a gift from Matthew Meyerson (Addgene plasmid #51070; http://n2t.net/addgene:51070; RRID:Addgene_51070), using LR clonase II mix (Invitrogen). *pDONR221_EGFP* (27), a gift from David Root (Addgene plasmid #25899; http://n2t.net/addgene:25899; RRID:Addgene_25899), was used to create *pBABEpuro-gateway-eGFP,* a control for the retroviral expression vector. Expression plasmids encoding ERBB mutants were generated by site-directed mutagenesis using the templates and primers listed in Supplementary Table S1. All the cloned constructs were verified by sequencing the insert.

For retroviral ERBB4 variant expression in BEAS-2B cells, expression constructs (Supplementary Table S1) were generated into *pMSCV-PGK-Puro-IRES-GFP* backbone [Murine Stem Cell Virus (MSCV) backbone (28)] using standard molecular techniques.

### Cell Culture

Phoenix-Ampho cells (a gift from Garry Nolan, not authenticated), Ba/F3 cells (a gift from David M. Weinstock, not authenticated), and BEAS-2B cells (ATCC CRL-9609, obtained in 2019, authenticated using short tandem repeat analysis) were cultured in RPMI1640 (Lonza or Gibco), supplemented with 10% FCS (Biowest), 50 U/mL penicillin and streptomycin solution (Lonza), and 2 mmol/L l-glutamine (Lonza). Unless otherwise stated, growth medium for Ba/F3 cells was supplemented with 5% conditioned medium from WEHI cells (as source of IL3). COS-7, NIH-3T3 (ATCC CRL-1658, obtained in 2019, not authenticated) and Platinum-E cells (Cell Biolabs #RV-101, obtained in 2012, not authenticated) were cultured in DMEM supplemented with 10% FCS, 50 U/mL penicillin and streptomycin solution and 2 mmol/L l-glutamine. Cells were routinely tested for *Mycoplasma* infection using MycoAlert (Lonza).

### Generation of Cell Lines with Stable Overexpression of ERBB4 Variants

To generate Ba/F3 and NIH-3T3 cell lines with stable overexpression of ERBB4 variants, Phoenix-Ampho packaging cells were transfected with retroviral *pBABEpuro-gateway* constructs encoding ERBB4 variants or enhanced GFP (eGFP) using FuGENE 6 transfection reagent (Promega) according to manufacturer's instructions. The retroviral supernatants of Phoenix-Ampho cells harvested 24 and 48 hours after transfection were incubated on Ba/F3 and NIH-3T3 cells for 6 hours on 2 consecutive days in the presence of 0.8 µg/mL hexadimethrine bromide (Polybrene, Sigma-Aldrich). Cell pools with stable expression were selected with 2 µg/mL puromycin (Gibco) and then maintained in 1 µg/mL puromycin for Ba/F3 cells, or with 6 µg/mL puromycin and then maintained in 3 µg/mL puromycin for NIH-3T3 cells.

To generate BEAS-2B cell lines with stable overexpression of ERBB4 variants, Platinum-E packaging cells were transfected with retroviral *pMSCV-PGK-Puro-IRES-GFP* constructs encoding ERBB4 variants or an empty vector using X-tremeGENE 9 transfection reagent (Roche) according to manufacturer's instructions. The retroviral supernatants of Platinum-E cells were harvested 48 hours after transfection and incubated on BEAS-2B cells for 72 hours. Transduced BEAS-2B cells were cultured in the presence of 1 µg/mL puromycin to select and maintain cell lines with stable expression.

### Generation of an Expression Library Encoding Random ERBB4 Mutations

An expression library for randomly mutated *ERBB4* cDNAs was generated by using GenemorphII random mutagenesis kit (catalog no. 200550; Agilent Technologies), as described previously (29). Briefly, an error-prone PCR reaction was carried out for 10 cycles using 6.08 µg of the plasmids *pDONR221-ERBB4JM-aCYT-1* and *pDONR221-ERBB4JM-aCYT-2* (equivalent to 4 µg of ERBB4 cDNA inserts) as templates, and the oligonucleotides 5′-ttgatgcctggcagttcccta -3′ (binds 78 bp upstream of attL1 site), and 5′- atcttgtgcaatgtaacatcagagatt-3′ (binds 80 bp downstream of attL2 site), as the primer pair, according to the instructions of GenemorphII random mutagenesis kit. The reaction produced 4,337 and 4,289 bp amplicons for mutated ERBB4 JM-a CYT-1 and ERBB4 JM-a CYT-2, respectively. The amplicons were run on agarose gel, purified with NucleoSpin Gel and PCR Clean-up kit (Macherey Nagel), and cloned into *pBABEpuro-gateway* (26), using LR clonase II mix (Invitrogen). The LR cloning product was transformed into *ccdb*-sensitive *E. coli* to create expression libraries for *ERBB4* JM-a CYT-1 and *ERBB4* JM-a CYT-2.

### *In Vitro* Screen for Activating ERBB4 Mutations

In the screen for activating ERBB4 mutations, we utilized the *in vitro* screen for activating ERBB4 mutations (iSCREAM) methodology, which we published earlier as a proof-of-concept study with EGFR (29). To generate Ba/F3 cells stably expressing the ERBB4-mutant libraries, 1 pmol of *pBABEpuro-gateway* JM-a CYT-1 or JM-a CYT-2 expression libraries were transfected to Phoenix-Ampho cells for retroviral production and infection of 5 × 10^5^ Ba/F3 cells as described above. To select for Ba/F3 cell clones expressing ERBB4 mutations providing growth advantage, 7.6 × 10^6^ of puromycin-resistant Ba/F3 cells were washed twice with 20 mL of PBS and maintained in 40 mL of RPMI1640 (containing 2 mmol/L l-glutamine, 50 U/mL penicillin-streptomycin, 10% FCS, and 1 µg/mL puromycin) in the presence or absence of 5% WEHI cell medium (as source or IL3) or 10 ng/mL neuregulin-1β (NRG-1β; R&D systems). The cells were passaged prior to reaching confluency to maintain growth in log-phase. Samples of cells expressing the ERBB4 JM-a CYT-2 mutation library, surviving in the presence of NRG-1 but absence of IL3, were frozen when passaging the cells and at the termination of the culture.

To determine the enrichment and depletion of specific ERBB4 variants upon clonal evolution of the Ba/F3 cells, genomic DNA was extracted from the frozen cell samples with NucleoSpin Tissue kit (Macherey-Nagel). The *ERBB4* cDNA variants were PCR-amplified (30 cycles) using 100 ng as a template with a 1:1 mixture of Phusion (Thermo Fisher Scientific) and Velocity (Bioline) high fidelity DNA polymerases and the primer pair: 5′-gaacctcctctttcgacccc-3′ and 5′-aagagttcttgcagctcggt-3′ which are complementary to the vector sequences flanking the *ERBB4*-mutant inserts. Amplicons were purified (NucleoSpin Gel and PCR Clean-up kit, Macherey-Nagel) and sequencing libraries were prepared with Nextera XT DNA Sample Preparation Kit (Illumina) according to manufacturer's instructions. Samples were sequenced on Illumina MiSeq with 150 bp paired-end sequencing producing on average 5.81 million reads per sample. Trimmed reads [trimmomatic (30)] were aligned to “hg19” human reference genome with BWA-MEM (v0.7.13-r1126). Variant calling and annotation was performed with samtools (v1.3.1) (31) and ANNOVAR (32), respectively. The variant frequency of a mutation was defined as the ratio of the number of reads with a particular mutation to the total number of reads aligned to the same locus. The fold change of a mutation at a particular timepoint was calculated by dividing the variant frequency of the mutation at a specific timepoint by the variant frequency of that mutation in the original plasmid library.

### Ba/F3 Cell Growth Assays

Ba/F3 cells were deprived of IL3 by washing twice with PBS and seeded at 1 × 10^5^ cells/mL in culture medium supplemented or not with 5% WEHI cell conditioned medium (source of IL3) or 10 ng/mL NRG-1. At the indicated timepoints, Ba/F3 cell viability was measured using the MTT assay (CellTiter 96 Nonradioactive Cell Proliferation Assay; Promega) by sampling 100 µL of the cell suspension into 96-well plates in quadruplicates. The growth curves were fitted using the linear-quadratic model and graphically displayed using GraphPad Prism 9 (GraphPad Software Inc.). The doubling time for cells growing in 10 ng/mL NRG-1 was calculated by the equation 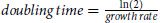, and 
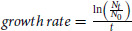
, where *t* is the time in reaching saturation density, *N_t_* is absorbance at saturation density, and *N*_0_ is the absorbance at seeding density. Statistical testing was carried out using, the Welch two sample *t* test. To correct for multiple testing, the FDR was controlled using the Benjamini–Hochberg procedure.

### Western Blotting

Cells were lysed in lysis buffer [1% Triton X-100, 10 mmol/L Tris-Cl, pH 7.4, 150 mmol/L NaCl, 1 mmol/L EDTA, 10 mmol/L NaF, supplemented with cOmplete Mini-EDTA free Protease Inhibitor Cocktail (Roche)]. Proteins in the samples were separated by SDS-PAGE and transferred to nitrocellulose membranes. The following primary antibodies were used: anti-β-actin (A5441; Sigma-Aldrich), anti-EGFR (#4267, Cell Signaling Technology), anti-ERBB2 MA5-14057, anti-ERBB3 (#4754, Cell Signaling Technology), anti-ERBB4 (clone E200, ab32375, Abcam), anti-phospho-ERBB4 (#4757, Cell Signaling Technology), and anti-GFP (ab183734, Abcam). The signals were detected using enhanced chemiluminescence and quantified by densitometry.

### Soft Agar Anchorage-Independent Growth Assay of NIH-3T3 Cells

Soft agar anchorage-independent growth assay was modified from a previously described protocol (33). Bottom layers (50 µL) consisting of 0.6% agar (ACROS Organics) in DMEM (supplemented with 10% FCS, 50 U/mL penicillin and streptomycin solution, 2 mmol/L l-glutamine, 2 µg/mL puromycin) were casted on 96-well plates. NIH-3T3 transductants were seeded as a middle layer on top of the solidified bottom layers in 96-well plates in quadruplicates (5,000 cells/well) as 10 µL cell suspensions mixed with 50 µL 0.4% agar in DMEM (supplemented as above, and with 50 ng/mL NRG-1). Semisolid feeder layers (50 µL) consisting of 0.6% agar in DMEM (supplemented as in bottom layers, and with 100 ng/mL NRG-1) were casted onto the solidified middle layer. After growing the cells for 11 days, cell viability was analyzed using alamarBlue (Invitrogen) according to manufacturer's instructions and the fluorescence intensity was measured 24 hours after adding the dye. Statistical analysis was performed by pairwise comparisons between cells expressing wild-type or mutant ERBB4 variants (Brown-Forsythe and Welch ANOVA tests, Dunnett T3 multiple comparisons test). *P* < 0.05 were considered as significant. GraphPad Prism 9 was used for creating the dot plot and performing the statistical analyses.

### Three-Dimensional Matrigel Growth Assay of BEAS-2B Cells

Five mg/mL of poly(2-hydroxyethyl methacrylate; poly-HEMA; Sigma-Aldrich) in 96% ethanol was used to coat 96-well plates (50 µL/well) for the three-dimensional (3D) growth assays. BEAS-2B cells stably expressing GFP-linked ERBB4 variants or GFP only from a bicistronic vector, were plated in quintuplicates on the poly-HEMA-coated 96-well plates (1,000 cells/well) in 2% Growth Factor Reduced Matrigel (Corning) in the presence of 2% FCS and 50 ng/mL NRG-1, and cultured for 7 days. 3D growth was assessed by subtracting the fluorescence intensity measured on the day of plating from the intensity measured seven days after plating. Statistically significant differences were tested by pairwise comparisons between cells expressing wild-type or mutant ERBB4 variants (Brown-Forsythe and Welch ANOVA tests, Dunnett T3 multiple comparisons test). *P* < 0.05 were considered as significant. GraphPad Prism 9 was used for creating the dot plot and performing the statistical analyses.

### *In Vivo* Allogenic Tumor Growth Assay

For the *in vivo* tumor growth assays, murine Ba/F3 cells (5 × 10^6^ cells in 100 µL PBS + 5% FCS) expressing ERBB4 variants were injected subcutaneously into the left and right flanks of female NMRI nude mice (BomTac:NMRI-Foxn1^nu^) ages 6–8 weeks. Tumor growth was monitored thrice weekly by bilateral caliper measurements and the tumor volume (*V*) was calculated using the formula: 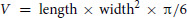. Tumor growth curves were plotted using the mean + SEM with Graphpad Prism 9.

Data showing the days until the tumors grew > 500 mm^3^ in volume are presented as a dot plot, from which tumors that did not reach 500 mm^3^ by day 60 were excluded. For statistical analysis, pairwise comparisons between the mutants and the wild-type were performed by Kruskal–Wallis test followed by Dunn multiple comparisons test. *P* < 0.05 was considered as significant. GraphPad Prism 9 was used for creating the dot plot and performing the statistical analyses. All animal studies were conducted in an Association for Assessment and Accreditation of Laboratory Animal Care International accredited animal facility at Boehringer Ingelheim, Austria in accordance with EU legislation and were approved by Austrian Authorities.

### Drug Sensitivity Assays

Ba/F3 cells (20,000 cells/well) expressing ERBB4 variants were plated on 96-well plates in RPMI1640 medium containing 10 ng/mL NRG-1. Vector control cells were plated in RPMI1640 medium containing 5% WEHI conditioned medium. The cells were incubated in the presence or absence of 0.0005–10 µmol/L erlotinib (Santa Cruz Biotechnology), afatinib (Boehringer Ingelheim), neratinib (SantaCruz Biotechnology), dacomitinib (Cayman Chemicals), poziotinib (Selleck Chemicals), ibrutinib (Selleck Chemicals), or lapatinib (Santa Cruz Biotechnology), for 72 hours before measuring the viability of cells with MTT assay (described above). Sigmoidal dose–response curves were fitted using asymmetric five-parameter non-linear regression in GraphPad Prism 9 and are graphically displayed with GraphPad Prism 9. The IC_50_ values are calculated with the help of the R package “drc” (34) by using quadruplicate measurements from the MTT assay for the indicated concentrations from 3–5 individual experiments and fitting four-parametric log-logistic models (LL.4, LL2.4; R). The unpaired *t* test (Welch two-sample *t* test) was used to compare IC_50_ values.

### Structure Preparation

All 3D structures were obtained from the Protein Data Bank [PDB (35)]. For ERBB4 R687K mutation study, the dimeric wild-type ERBB4 transmembrane (TM)-(JM) domain was first modeled on the basis of the respective NMR structure from EGFR [PDB ID: 2M20 (36); model 1—the best representative conformer in the ensemble] using Modeler (37). The TM domain of the resulting model was subsequently replaced by the NMR structure of ERBB4 TM domain [PDB ID: 2LCX (38); model 1] in Chimera (39). The analysis of the ERBB4 E715K mutation was based on the 2.5 Å X-ray structure of the intracellular ERBB4 asymmetric kinase domain dimer [PDB ID: 3BCE (40); symmetry operations were employed with Chimera to attain the asymmetric dimer orientation of the structure]. On the basis of each of the above wild-type structures, the R687K- and E715K-mutant forms were built by simply making the amino acid replacements using Chimera. The four wild-type and mutant ERBB4 structures were then prepared with the protein preparation wizard in Maestro (41) by adding hydrogen atoms, optimizing hydrogen bonds, determining protonation states of ionizable side chains and energy minimizing the structures.

### Molecular Dynamics Simulations

Molecular dynamic simulations (MDS) were used to probe the dynamic structural effect of the mutations by comparing the prepared wild-type and mutant ERBB4 structures simulated with the Amber program (ref. 42; version 2018), and using the ff14SB (43) and LIPID17 force fields respectively for proteins and lipids. The wild-type and E715K ERBB4 asymmetric kinase domain were solvated in an octahedral box with the TIP3P water model (44) with a 12 Å distance between the solute atoms and the box periphery. The simulation systems for the wild-type and R687K ERBB4 TM-JM dimer structures were built using the CHARMM-GUI web server (45). Membrane bilayers were built composed of 100% POPC (1-palmitoyl-2-oleoyl-*sn*-glycero-3-phospho-l-serine) on the upper leaflet (extracellular facing) and 70% POPC with 30% POPS (1-palmitoyl-2-oleoyl-*sn*-glycero-3-phospho-l-serine) on the lower leaflet. These two systems were solvated with TIP3P waters in a rectangular box (88 Å × 88 Å × 122 Å). All simulation systems were then neutralized by adding Na^+^ ions with additional Na^+^/Cl^−^ ions incorporated to attain 0.15 mol/L salt concentration.

Before executing the production simulations, the systems were minimized, heated and equilibrated. The equilibration protocol employed for the intracellular kinase domain systems is discussed in detail in ref. 46. The membrane-bound systems were equilibrated using the default protocol in the CHARMM-GUI, with 5,000 cycles of steepest descent and conjugate gradient energy minimization and a 10 and 2 kcal mol^−1^Å^−2^ restraint on protein and lipid atoms, respectively. This was followed by heating of the systems to 303.15 K and a five-stage equilibration with gradual reduction of restraints on solute atoms. The production simulations for all systems were performed for 100 ns at constant temperature (300 K/303.15 K) and pressure (1.0 bar). Coordinates were saved every 20 ps and analysis of the resulting trajectories was carried out using the programs CPPTRAJ (47) and VMD (48).

Root-mean-square deviations (RMSD) were computed on the basis of backbone heavy atoms relative to the starting structure. Hydrogen bonds were examined by defining a bond distance of ≤ 3.5 Å and a bond angle of ≥ 135°. Binding energy calculations were carried out using the molecular mechanics generalized Born surface area (MM-GBSA) program (49) in Amber. The solvent accessible surface area (SASA) for selected atoms was computed in CPPTRAJ with the linear combinations of pairwise overlaps (LCPO) method (50) using a probe radius of 1.4 Å. The helical content of the JM C-terminal helix was determined in Amber as the fraction of residues observed to remain helical during the simulation.

### Transactivation Assay

COS-7 cells were plated on 6-well plates and transiently transfected with the indicated pcDNA3.1 constructs encoding hemagglutinin (HA)-tagged wild-type, kinase-dead or kinase dimerization interface mutant ERBB receptors. One µg of plasmid DNA was used for single transfections and 0.5 µg + 0.5 µg for co-transfections with Lipofectamine2000 (Invitrogen) according to manufacturer's instructions. Cells were lysed 24 hours after transfection and analyzed for basal ERBB4 phosphorylation by Western blotting. The signals were detected using near-IR fluorescence with the Odyssey CLx imaging system (LI-COR). Pairwise comparisons of the data were made separately for each co-transfected ERBB by one-sample *t* test from log-transformed data.

### Data Availability Statement

Raw next-generation data were generated in a core facility and are available from the corresponding author on request. The processed data are available from the corresponding author upon request.

## Results

### Generation of cDNA Libraries for Expression of Randomly Mutated Single-Nucleotide *ERBB4* Variants

To screen for activating ERBB4 mutants in an unbiased manner, expression libraries of randomly mutated *ERBB4* cDNAs were created. Random mutations were generated by error-prone PCR using full-length cDNAs encoding the wild-type human ERBB4 isoforms JM-a CYT-1 or JM-a CYT-2 as templates, as described in our previous work with EGFR (29). These two ERBB4 isoforms were selected for the experimentation as they represent the isoforms present in epithelial-derived cancer tissues (51–53). The amplicons were subsequently cloned into a *pBABE-gateway* retroviral mammalian expression vector. Sanger sequencing demonstrated that an average mutation frequency of 2.11 or 2.59 mutations per 3,927 or 3,879 bp *ERBB4* cDNA inserts was obtained for the mutation libraries encoding variants of ERBB4 JM-a CYT-1 or JM-a CYT-2, respectively. The mutation frequency was comparable with that observed in our earlier analysis with an *EGFR* cDNA mutation library (2.67 mutations per cDNA), generated using the same conditions for the error-prone PCR (29).

### Setting Up a Functional Genetics Screen for Activating ERBB4 Variants in Ba/F3 Cells

To address the optimal conditions for a functional screen, the two expression libraries were transduced into murine lymphoid Ba/F3 cells that are dependent on exogenous IL3 for survival (54) but can be rendered IL3-independent by ectopic expression of oncogenic variants of a number of tyrosine kinases, including the ERBB receptors (55, 56). After transduction, the Ba/F3 cells were cultured in puromycin for 48 hours to select for cells with stable expression. Neither the Ba/F3 cells expressing wild-type ERBB4 isoforms, nor the respective mutant libraries, survived the withdrawal of IL3 (Supplementary Fig. S2A). However, cells expressing the ERBB4 JM-a CYT-2 mutation library demonstrated robust cell growth in the presence of exogenous NRG-1, outperforming the growth promoted by the wild-type ERBB4 JM-a CYT-2 (Supplementary Fig. S2B), suggesting the presence of activating ERBB4 variants in the cell pool. In contrast, consistent with a previous report (57), Ba/F3 cells expressing the ERBB4 JM-a CYT-1 mutation library or wild-type ErbB4 JM-a CYT-1 did not demonstrate NRG-dependent growth within the first 5 days after plating (Supplementary Fig. S2C). This difference in the activity of the two ERBB4 CYT isoforms in another IL3-dependent murine cell model, the 32D cells, has been reported before (24). On the basis of these initial validation data, the ERBB4 JM-a CYT-2 mutation library and culture conditions including exogenous ligand were chosen for the functional screen.

### ERBB4 Mutations in the Expression Library

To characterize the presence and distribution of ERBB4 variants in the ERBB4 JM-a CYT-2 mutation library, the *ERBB4* insert was PCR amplified from the plasmid library with primers specific to the retroviral vector backbone and deep sequenced (>100,000 X) on Illumina MiSeq platform. Sequencing data demonstrated that the mutant ERBB4 JM-a CYT-2 library harbored 8,386 unique *ERBB4* variants at the nucleotide level out of the 11,638 theoretically possible variants (72.1%). Inference of the resulting amino acid alterations indicated the presence of 7,539 of the 8,065 theoretically possible changes, yielding a coverage of 93.5% of all possible ERBB4 missense or nonsense mutations. The individual mutations in the library were relatively evenly distributed into different transition and transversion mutations (Supplementary Fig. S3A) as well as into specific missense or nonsense mutations (Supplementary Fig. S3B), with the exception of the lack of mutations altering the stop codon in wild-type *ERBB4*.

### Functional Screen and Deep Sequencing of ERBB4 Variants

To select for activating ERBB4 variants that enhance IL3-independent proliferation of Ba/F3 cells, cells overexpressing the mutant expression library or wild-type ERBB4 were maintained in 10 ng/mL NRG-1 in the absence of IL3. The cells were maintained in a phase of exponential growth by passaging prior to reaching confluency. The experiment was terminated on day ten when the rate of proliferation of the cells expressing wild-type ERBB4 started to match that of the cells expressing the mutation library (Supplementary Fig. S2B). The sample of surviving cells frozen at day 8 was chosen for analysis to represent a pool of mutant ERBB4-expressing cells proliferating at a higher rate than wild-type ERBB4-expressing cells.

Genomic DNA was extracted from the surviving cells and the *ERBB4* cDNA inserts integrated into the Ba/F3 cell genomes were PCR amplified and deep sequenced similarly as described above for the original cDNA library. The number of ERBB4 coding sequence variants that were present in the sequencing data of both the samples representing the original cDNA library and the evolved Ba/F3 cell pool was 7,396, which constitutes a 91.7% coverage of the 8,065 theoretically possible *ERBB4* alterations, and 98.1% of the 7,539 alterations present in the cDNA library.

### ERBB4 Variants Promoting Clonal Selection of Ligand-Dependent Ba/F3 Cells

**FIGURE 1:**
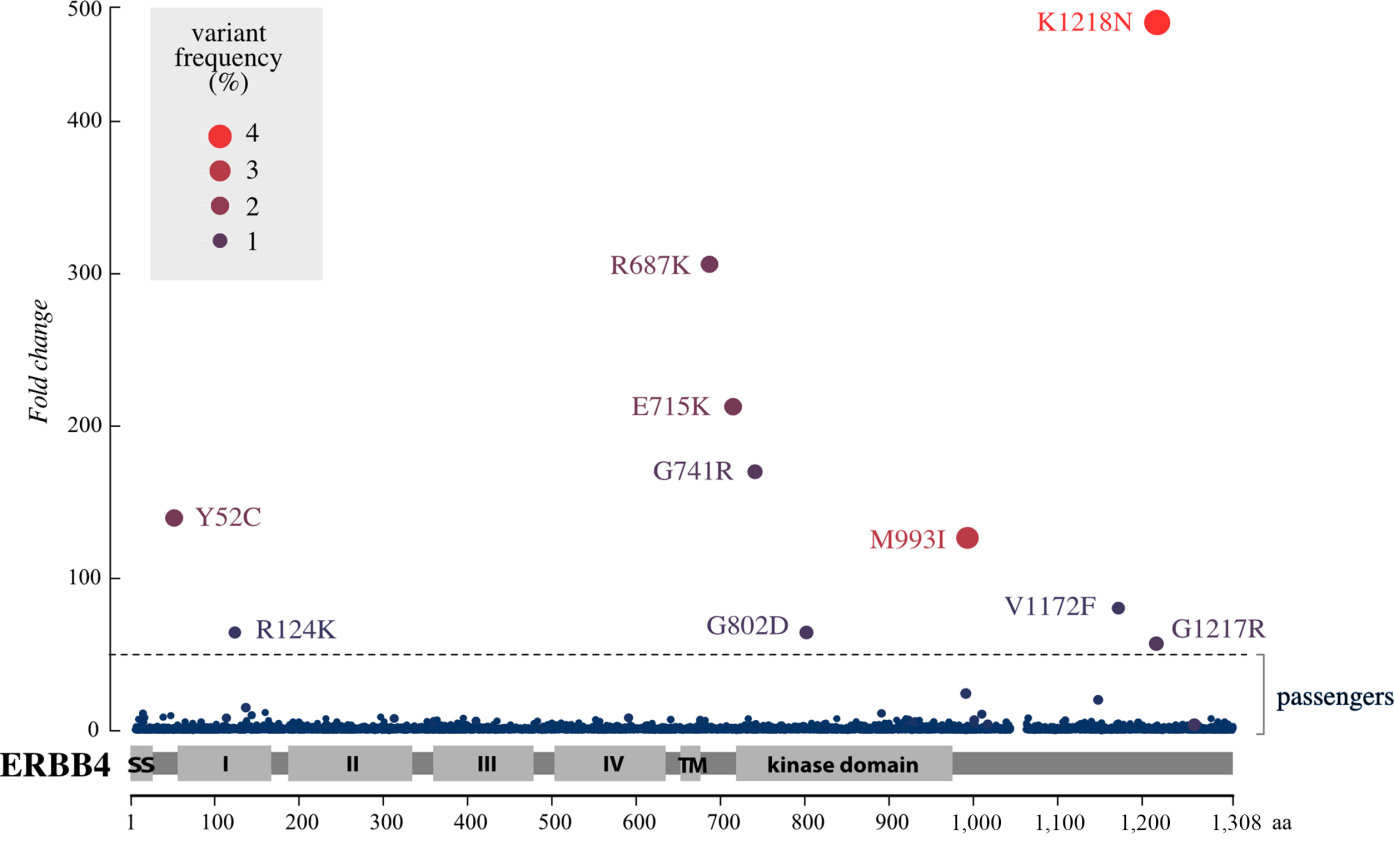
iSCREAM of ERBB4. Scatter plot demonstrates the enrichment of specific ERBB4 mutations in Ba/F3 cells engineered to express 7,396 different missense and nonsense mutations of ERBB4. The cells were cultured for 8 days in the presence of 10 ng/mL of the ERBB4 ligand NRG-1 in the absence of IL3. The *x*-axis shows the position of the mutated residue on the ERBB4 primary sequence. The *y*-axis indicates the observed fold change in the variant frequency of a mutation in the surviving cell pool when compared with the variant frequency of that mutation in the cDNA mutation library. The dashed horizontal line indicates a cutoff at 50-fold enrichment (*q* < 0.00001) for the mutation in the surviving cell pool as compared with the mutation library. The size of the dot as well as the intensity of its red color indicate the variant frequency of the mutation in the surviving cell pool. The gap in the *x*-axis (at residues 1046–1061) represents the 16 amino acids (aa) that are absent in the ERBB4 splice variant JM-a CYT-2 which was used for generating the mutation library.

The comparison of the read counts specific for each ERBB4 coding sequence variant (normalized to total number of reads at each *ERBB4* locus) in the surviving cell population at day 8 versus the original cDNA library enabled estimation of the enrichment of each specific mutation (fold change) as well as the relative variant frequency of each mutation in the surviving cell pool (Fig. 1). The great majority of the assessed mutations (*n* = 7,386; 99.86%) demonstrated a nonsignificant enrichment indicating lack of growth-promoting activity and were called passenger mutations (Fig. 1). In contrast, 10 mutations were significantly (*q* < 0.00001) enriched (57- to 464-fold) in the surviving cells and were hypothesized to represent activating mutations contributing to growth and survival of the cells. These 10 mutations were distributed within the extracellular domain (Y52C and R124K), the JM segment of the intracellular domain (R687K and E715K), the kinase domain (G741R and G802D), and at the C-terminal tail (M993I, V1172F, G1217R, and K1218N). Although previously reported as variants of unknown function (https://cbioportal.org), interestingly, all the 10 amino acid positions identified as hits from the screen have been shown to be mutated in clinical cancer tissue samples (Supplementary Fig. S4).

### Functional Validation of Single ERBB4 Variants in Ba/F3 Cells

**FIGURE 2:**
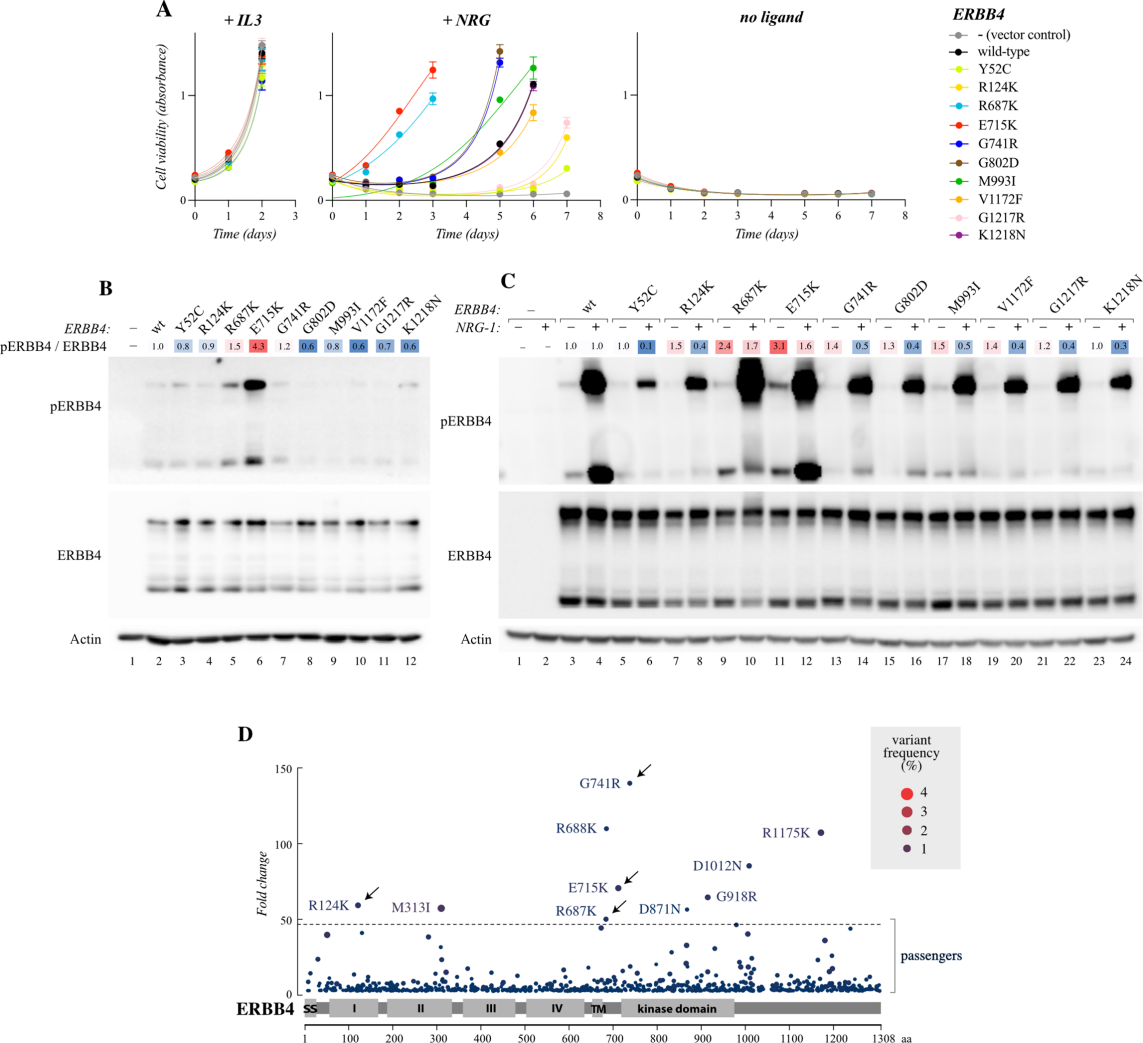
Validation of the screen in Ba/F3 cells. **A,** Ba/F3 cells expressing the indicated ERBB4 JM-a CYT-2 variants or vector control cells (−) were cultured in the presence or absence of IL3 or 10 ng/mL NRG-1. Cell viability was analyzed with the MTT assay. The mean and SD of quadruplicate analyses are shown. **B,** Western analysis of Ba/F3 cells expressing the indicated ERBB4 variants. The cells were cultured in the presence of IL3. wt, wild-type. Heatmap indicates fold changes in ERBB4 phosphorylation normalized to ERBB4 expression. **C,** Western analysis of Ba/F3 cells expressing the indicated ERBB4 variants. Cells were stimulated or not with 50 ng/mL NRG-1 in the absence of IL3 for 10 minutes. Heatmap indicates fold changes in ERBB4 phosphorylation normalized to ERBB4 expression. **D,** Scatter plot of an independent *in vitro* screen of activating ERBB4 mutations (iSCREAM) demonstrating the enrichment of specific mutations in Ba/F3 cells engineered to express 7,124 different missense and nonsense mutations of ERBB4. The cells were cultured for 10 days in the presence of 10 ng/mL NRG-1 but in the absence of IL3. The dashed horizontal line indicates a cutoff at 47-fold enrichment (*q* < 0.00001) for the mutation in the surviving cell pool as compared with the mutation library. Please see legend of Fig. 1 for the description of the axes and symbols. ERBB4 mutations were analyzed for ERBB4 phosphorylation in two to 10 independent experiments.

As the mutation frequency exploited in the error-prone PCR process produced mutations at an average rate of 1 change per 1,496 bases, equaling to 1.79 coding sequence variants per ERBB4 polypeptide, the clonal evolution of Ba/F3 cells could have favored enrichment of cDNAs harboring multiple *ERBB4* mutations *in cis*. To control for this, the top 10 mutations from the *ERBB4* mutation screen (Fig. 1) were selected for *in vitro* validation. Ba/F3 cells were transduced with retroviral constructs encoding individual ERBB4 mutations Y52C, R124K, R687K, E715K, G741R, G802D, M993I, V1172F, G1217R, K1218N, as well as wild-type ERBB4 JM-a CYT-2 or eGFP (as control). Ba/F3 cells stably expressing these constructs were tested for their potential to promote IL3-independent growth using the MTT assay (Fig. 2A). Five of the 10 mutations (R687K, E715K, G741R, G802D, and M993I) promoted IL3-independent growth in the presence of NRG-1 (10 ng/mL) to a greater extent than the cells expressing wild-type ERBB4 (*q* < 0.001; Supplementary Fig. S5A). These results indicate that the screen could identify activating *ERBB4* mutations that enhance ligand-dependent cell proliferation. To control that the NRG-dependent growth of the lines was dependent on ERBB4 expression as opposed to an unknown oncogenic integration of the retrovirus, the ectopic ERBB4 expression was knocked down from the cells by RNAi. The NRG-dependent growth was indeed efficiently suppressed by ERBB4-targeted short hairpin RNA (shRNA) but not by scrambled shRNAs (Supplementary Fig. S6).

To control for ERBB4 expression and autophosphorylation levels in the Ba/F3 cells expressing the ERBB4 variants, Western analyses were carried out for cells maintained in the presence of IL3 (Fig. 2B). The two mutations most efficient in promoting NRG-dependent Ba/F3 cell growth (R687K and E715K) were also found to be more phosphorylated than wild-type ERBB4, even under the conditions lacking selection pressure from IL3 withdrawal (Fig. 2B; Supplementary Fig. S5B). In particular, the E715K variant demonstrated robust ERBB4 autophosphorylation, suggesting that the E715K mutation renders the receptor constitutively active. Consistently, in Ba/F3 cells stimulated with 50 ng/mL NRG-1 (in the absence of IL3) for 10 minutes, both the basal and NRG-stimulated phosphorylation levels of ERBB4 R687K and E715K were greater in comparison with the wild-type ERBB4 (Fig. 2C). Of note, upon NRG-1 stimulation, the 180 kDa full-length ERBB4 R687K was more heavily phosphorylated than wild-type ERBB4 or ERBB4 E715K, whereas the 75 kDa intracellular domain (ICD) of ERBB4 R687K was appreciably less phosphorylated than the ICDs of the wild-type ERBB4 or ERBB4 E715K (Fig. 2C). The soluble 75 kDa ERBB4 ICD is an active tyrosine kinase produced upon two proteolytic cleavages of ERBB4 (58–60). It is worth noting, however, that the experimentation was carried out with an antibody recognizing the single phosphorylated tyrosine 1284 of ERBB4 and may thus not fully reflect all functional signaling downstream of an activated ERBB4 receptor (61).

Finally, to assess the reproducibility of the screen, we repeated the iSCREAM experiment (Fig. 2D) with an independently generated *ERBB4* random mutation library. This second ERBB4 iSCREAM experiment covered 7,124 amino acid substitutions (88.3% of the theoretical maximum). The two independent screens shared four common mutations among their respective lists of top 10 mutations (*q* < 0.00001). These common mutations were R124K, R687K, E715K, and G741R (annotated with arrows in Fig. 2D). Notably, three of these mutations (R687K, E715K, G741R) were also able to confer NRG-dependent growth advantage to the Ba/F3 cells (Fig. 2A). Collectively, these findings indicate that the screen is reproducible and can identify activating *ERBB4* mutations.

### Functional Validation of Single ERBB4 Variants in NIH-3T3 Cells

The ability of the top 10 identified ERBB4 mutations (identified in the first screen) to promote anchorage-independent growth was tested in an adherent cell model, NIH-3T3 mouse fibroblasts. In the presence of 50 ng/mL NRG-1, four mutants (R687K, E715K, G802D, and M993I) promoted anchorage-independent growth of NIH-3T3 significantly more than wild-type ERBB4 (Supplementary Fig. S7A). This is in line with the results in Ba/F3 cells, in which all of these four mutants (R687K, E715K, G802D, and M993I), in addition to G741R, promoted IL3-independent growth in the presence of NRG-1. Western analysis of the transduced NIH-3T3 cells indicated that ERBB4 R687K, E715K, G741R, and G802D were also more phosphorylated than wild-type ERBB4 (Supplementary Fig. S7B and C) when stimulated with 0, 10, or 50 ng/mL NRG-1. In contrast, the activation of ERBB4 M993I was comparable with the wild-type receptor (Supplementary Fig. S7B and C), even though the M993I-mutant cells outperformed cells expressing wild-type ERBB4 in the anchorage-independent growth assay (Supplementary Fig. S7A). However, in NIH-3T3 cells subjected to serum starvation for 1 or 3 days, all the 10 mutants demonstrated greater tyrosine phosphorylation levels than the wild-type receptor (Supplementary Fig. S7D).

### ERBB4 Mutations are Transforming in Non-Tumorigenic Human Lung Epithelial Cells

To test the activity of the ERBB4 mutations in a more relevant cellular context, the four common mutations identified in both of the independent screens ERBB4 R124K, R687K, E715K, and G741R, were assessed for their capacity to transform non-tumorigenic BEAS-2B human lung epithelial cells. Unlike the lymphoid Ba/F3 cells, epithelial cells often endogenously express ERBB4, notably its JM-a CYT-1 and JM-a CYT-2 isoforms at comparable levels (52). While the JM-a CYT-2 isoform of ERBB4 was chosen to conduct the screen in Ba/F3 cells, ERBB4 JM-a CYT-1 isoform has been suggested to be more oncogenic in some epithelial cell contexts (53, 62). Therefore, we chose to use the JM-a CYT-1 isoform in the BEAS-2B experiments. To control for the use of a different isoform than the one used in the Ba/F3 experiments, the most activating mutation, E715K, was analyzed in the context of both CYT isoforms in the BEAS-2B cells. In addition, a previously characterized activating ERBB4 mutation, K935I (14), was included as a positive control.

**FIGURE 3:**
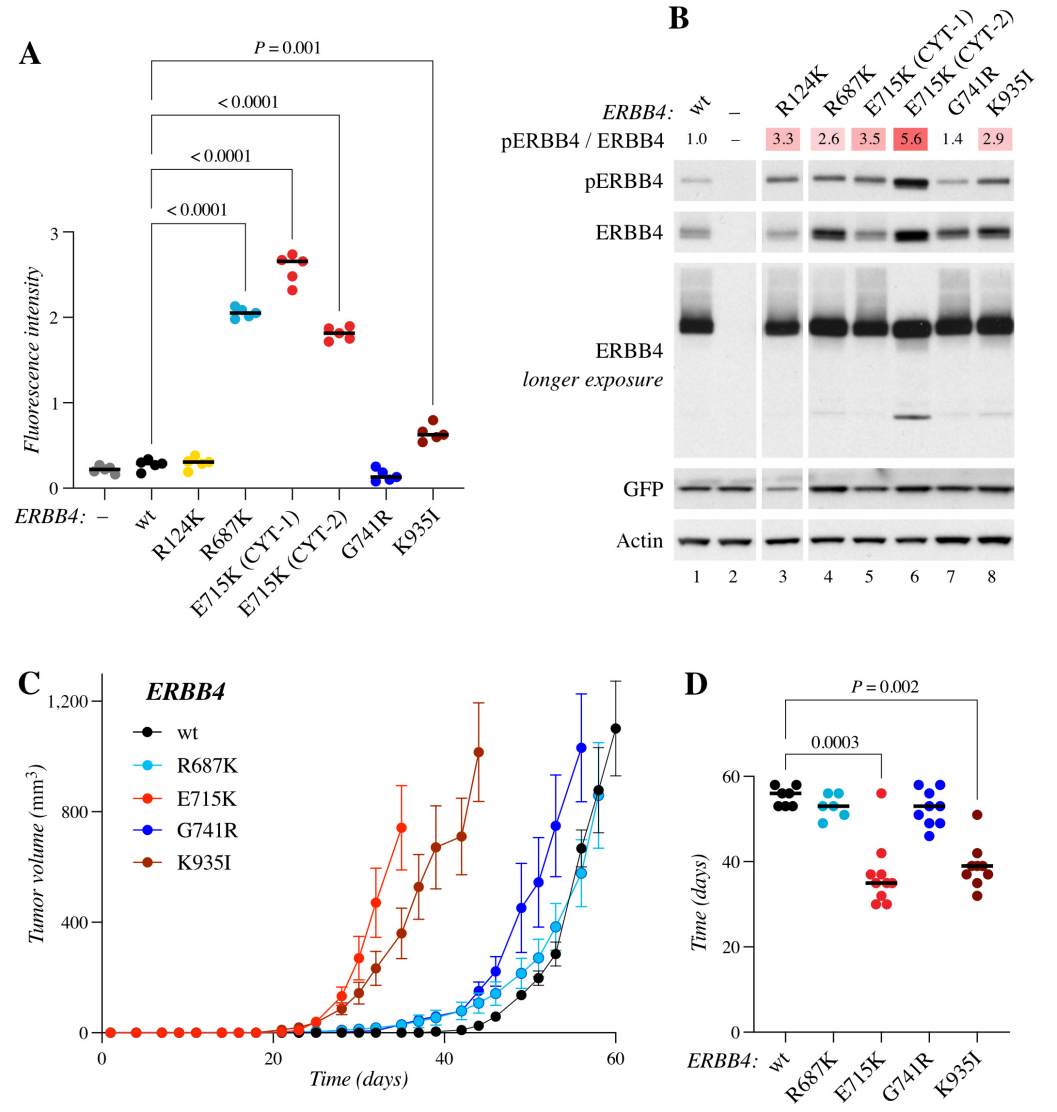
Activity of ERBB4 variants in promoting 3D and *in vivo* tumor growth. **A,** 3D growth of BEAS-2B cells expressing the indicated ERBB4 JM-a CYT-1 variants, or JM-a CYT-2 E715K, or vector control cells (−) *in vitro*. The cells were cultured in Matrigel for 7 days in a medium containing 2% FCS and 50 ng/mL NRG-1. Fluorescence was measured from quintuplicate samples on days 0 and 7. Statistically significant *P* values (*P* < 0.05) for pairwise comparisons between the wild-type and the other cell lines are shown (Brown-Forsythe and Welch ANOVA, Dunnett T3 multiple comparisons test). Dots represent the technical replicates, black horizontal lines denote medians. Data shown are representative of four independent experiments. **B,** Western analysis of BEAS-2B cells expressing the indicated ERBB4 JM-a CYT-1 variants or ERBB4 JM-a CYT-2 E715K. Cells were maintained in the presence of 10 ng/mL NRG-1. Heatmap indicates fold changes in ERBB4 phosphorylation normalized to ERBB4 expression. Data shown are representative of two independent experiments. **C,** Growth of subcutaneous mouse allografts of Ba/F3 cells expressing the indicated ERBB4 variants. **D,** Dot plot indicating the days for the Ba/F3 allograft tumors (shown in **C**) to reach a volume > 500 mm^3^. Dots represent individual tumors, black horizontal lines denote medians; tumors that did not reach 500 mm^3^ by day 60 were excluded. Statistically significant pairwise comparisons (*P* < 0.05) between the wild-type and the mutants are shown (Kruskal–Wallis test, Dunn multiple comparisons test). wt, wild-type.

To assess the transforming potential of the activating ERBB4 mutations in 3D culture, the BEAS-2B cells stably expressing the ERBB4 mutants were seeded in 2% Matrigel on poly-HEMA–coated plates (to prevent adherence to the cell culture plate) and were allowed to grow for 7 days in the presence of NRG-1. The cells overexpressing wild-type ERBB4 did not promote 3D growth over the BEAS-2B cells transduced with a vector control (Fig. 3A). This indicates that a mere overexpression of wild-type ERBB4 is not sufficient to transform the BEAS-2B cells. In contrast, the cells expressing the ERBB4-mutants R687K, E715K, and the positive control K935I significantly promoted 3D growth of BEAS-2B cells (Fig. 3A), confirming transforming potential of these mutants also in the context of non-transformed human epithelial cells. In addition, the ERBB4 JM-a CYT-1 E715K was significantly more potent in promoting 3D growth of BEAS-2B cells (*P* = 0.002) than the JM-a CYT-2 E715K (Fig. 3A), supporting the previous findings that ERBB4 CYT-1 isoforms are potentially more transforming than CYT-2 isoforms in epithelial cells (53, 62). In contrast, the other two mutants identified in both screens, R124K and G741R, were unable to transform the BEAS-2B cells. Western analysis demonstrated that all the analyzed ERBB4 mutants apart from G741R exhibited higher levels of tyrosine phosphorylation than wild-type ERBB4 when the BEAS-2B cells were cultured in the presence of 10 ng/mL NRG-1 and 10% FCS (Fig. 3B).

### ERBB4 Mutations Promote Tumor Growth *In Vivo*

To study the tumorigenicity promoted by the ERBB4 mutations *in vivo*, subcutaneous Ba/F3 allografts were established in NMRI nude mice from cells overexpressing wild-type ERBB4, the ERBB4 variants R687K, G741R, or E715K, or the positive control ERBB4 K935I. The growth kinetics of the tumors expressing ERBB4 E715K or the positive control K935I were significantly faster than those expressing wild-type ERBB4 or the R687K or G741R mutants (Fig. 3C and D). While the tumors expressing ERBB4 G741R grew marginally faster than the tumors expressing wild-type ERBB4, this comparison did not reach statistical significance (Fig. 3D). Cells overexpressing the ERBB4 R124K mutant were also analyzed but, as the developed tumors demonstrated undetectable ERBB4 mRNA expression (Supplementary Fig. S8), the R124K data were removed from further analyses.

### Cells Expressing Mutant ERBB4 Receptors are Sensitive to pan-ERBB Inhibitors

**FIGURE 4:**
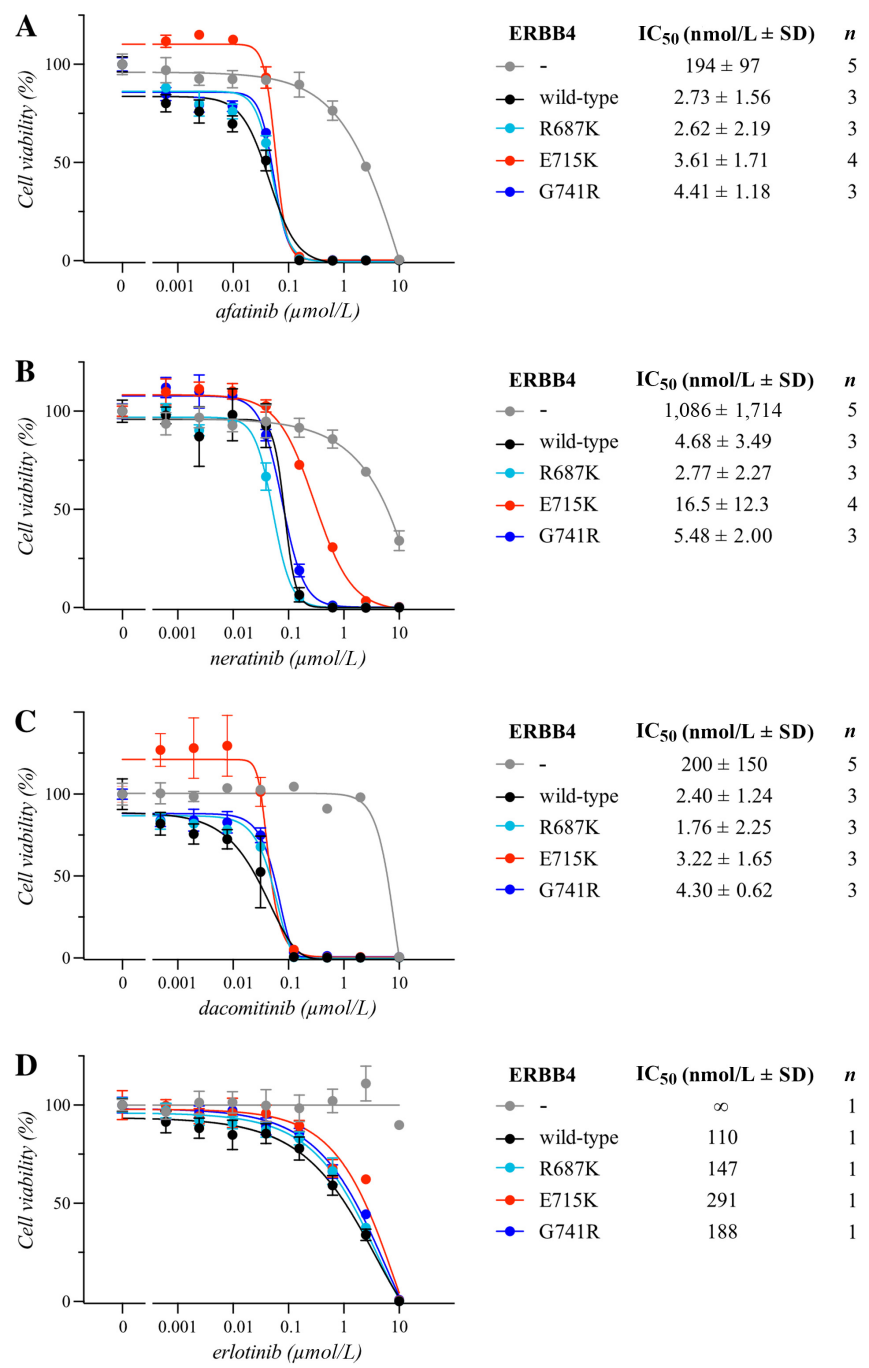
Sensitivity of ERBB4 mutations to tyrosine kinase inhibitors. Ba/F3 cells expressing the indicated ERBB4 variants cultured for 3 days in the presence of the indicated concentrations of afatinib (**A**), neratinib (**B**), dacomitinib (**C**), or erlotinib (**D**). Cells expressing ERBB4 variants were cultured in the presence of 10 ng/mL NRG-1 in IL3-free medium. The vector control cells were cultured in the presence of IL3. Cell viability was measured with the MTT assay. The mean and SD of quadruplicate analyses are shown for the dose–response curves. IC_50_ values for the drug responses were calculated from the indicated number of independent analyses (*n*) after fitting the dose–response curves with four-parameter log-logistic function (R; ‘drc’ package).

We also assessed potential means to pharmacologically target the identified activating ERBB4 mutations. Several small-molecule TKIs targeting the ERBB family have been developed and are clinically available (1). From these, the second-generation pan-ERBB TKIs, including afatinib, dacomitinib, and neratinib, also directly target ERBB4 (16). Thus, we assessed the potency of afatinib, dacomitinib, and neratinib to inhibit the ERBB4 mutation–dependent cell proliferation in Ba/F3 cells. The NRG-dependent growth promoted by the three mutations (R687K, G741R, E715K) was readily inhibited by afatinib with IC_50_ values in the low nanomolar range (Fig. 4A–C). Similarly, dacomitinib and neratinib inhibited NRG-dependent growth of the four mutant cell lines whereas erlotinib, an EGFR-selective TKI, did not (Fig. 4D). No significant differences were observed between cells expressing the ERBB4 mutants versus the wild-type receptor for any of the three TKIs (*q* > 0.5 for all comparisons), similar to what has previously been reported for predictive ERBB mutations such as EGFR L858R when compared with wild-type EGFR (17). Together, these results demonstrate that the iSCREAM pipeline can identify novel activating ERBB4 mutations that are transforming *in vitro* and *in vivo*, and that these mutants are sensitive to clinically used pan-ERBB inhibitors.

### Constitutively Active ERBB4 E715K Promotes Ligand-Independent Proliferation

**FIGURE 5:**
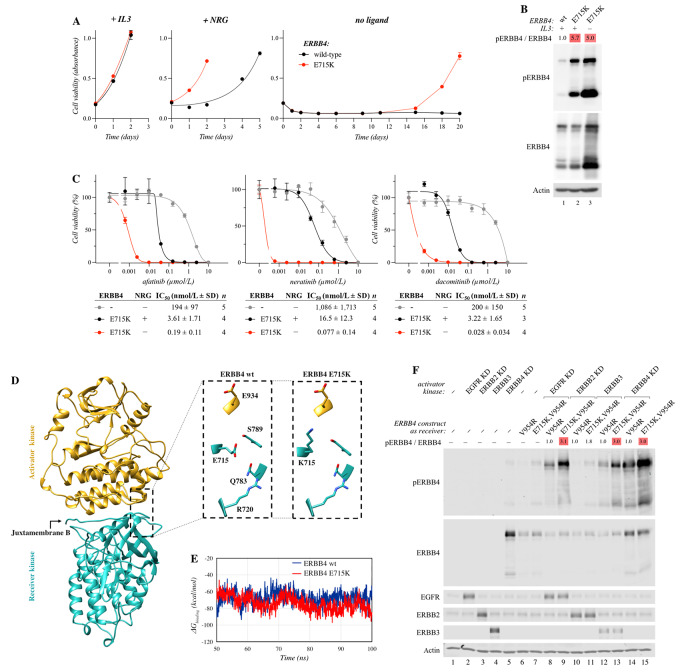
ERBB4 E715K. **A,** Ba/F3 cells expressing wild-type ERBB4 or ERBB4 E715K were cultured in the presence or absence of IL3 or 10 ng/mL NRG-1. Cell viability was analyzed with the MTT assay. The mean and SD of quadruplicate analyses are shown. **B,** Western analysis of Ba/F3 cells expressing wild-type (wt) ERBB4 or ERBB4 E715K maintained in the presence or absence of IL3. Heatmap indicates fold changes in ERBB4 phosphorylation normalized to ERBB4 expression. Data shown are representative of four independent experiments. **C,** Sensitivity of ligand-dependent and -independent Ba/F3 cell clones expressing ERBB4 E715K to the indicated tyrosine kinase inhibitors. Vector control cells (−) were cultured in the presence of IL3. Cell viability was measured with MTT assays. The mean and SD of quadruplicate analyses are shown for the dose–response curves. IC_50_ values for the drug responses were calculated from the indicated number of independent analyses (*n*) after fitting the dose–response curves with four-parameter log-logistic function (R; `drc' package). **D,** The ERRB4 activator (gold) and receiver (cyan) kinase domains with a focus on the location of E715 and E715K in the wild-type (wt) and mutant ERBB4 structures. **E,** Free energy of binding between the activator and receiver kinase domains of the wild-type (wt) and E715K-mutant ERBB4 structures during the simulations. **F,** Transactivation assay to address the activity of ERBB4 E715K as the receiver kinase (i) in heterodimers with wild-type or kinase-dead (KD) EGFR, ERBB2, ERBB3, or (ii) as a homodimer with kinase-dead ERBB4. In the assay, kinase-dead forms exclusively act as the activator kinases while wild-type ERBB3 is naturally kinase-dead. The ERBB4 E715K mutation was introduced into ERBB4 V954R mutant background because the V954R mutants can exclusively function as the receiver kinase. The assay was carried out in COS-7 cells transiently expressing the indicated constructs. Western analysis of ERBB4 phosphorylation was used as a readout for ERBB4 activation. Heatmap indicates fold changes in ERBB4 phosphorylation normalized to ERBB4 expression. Data shown are representative of four to six independent experiments.

While most of the studied ERBB4 mutants did not support IL3-independent proliferation of Ba/F3 cells in the absence of NRG-1 (Fig. 2A), a completely NRG-independent population evolved from cells expressing the E715K mutation after sustained culture in growth medium completely devoid of exogenous IL3 and NRG-1 (Fig. 5A). This is consistent with the observations that the E715K mutation demonstrated the highest autophosphorylation levels of all the ERBB4 mutants analyzed in all cell backgrounds tested (Fig. 2B and C; Fig. 3B; Supplementary Fig. S7B–D), suggesting that the mutation renders ERBB4 constitutively active. Interestingly, no similar NRG-independent clones emerged from the cells expressing R687K or G741R. These findings are consistent with the observation that the E715K variant was more potent in the *in vivo* allograft tumor growth assay (Fig. 3C and D) that was also carried out under conditions in which no exogenous NRG-1 was administered.

ERBB4 receptor expression was upregulated in the NRG-independent E715K cell population compared with E715K cells maintained in the presence of IL3 (Fig. 5B). Especially, the 75 kDa ICD was more abundant and prominently phosphorylated in the NRG-independent Ba/F3 cells harboring ERBB4 E715K. To address whether the upregulation of ERBB4 expression affected the sensitivity of the cells to pan-ERBB TKIs, drug response assays with afatinib were carried out in parallel with the NRG-dependent and -independent cells expressing ERBB4 E715K (Fig. 5C). The NRG-independent ERBB4 E715K cells had significantly (*P* = 0.013) lower IC_50_ for afatinib (0.19 ± 0.11 nmol/L), as compared with the NRG-dependent ERBB4 E715K cells (IC_50_ = 3.61 ± 1.71 nmol/L). The NRG-independent E715K cells also had statistically significantly lower IC_50_ for dacomitinib (IC_50_ = 0.028 ± 0.034 nmol/L; *P* = 0.039) and neratinib (IC_50_ = 0.077 + 0.14 nmol/L; *P* = 0.038) in comparison with NRG-dependent E715K cells (IC_50_ dacomitinib = 3.22 ± 1.65 nmol/L; IC_50_ neratinib = 16.5 ± 12.3 nmol/L; Fig. 5C). In addition to these second-generation TKIs, the NRG-independent cells expressing ERBB4 E715K mutant also demonstrated sensitivity to poziotinib and ibrutinib but not to lapatinib (Supplementary Fig. S9).

### E715K Mutation Enhances Kinase Transactivation in ERBB Homodimers and Heterodimers

To get insight into the molecular mechanism by which the E715K mutation increases ERBB4 activity, we performed *in silico* structural analyses for E715K. Glutamate 715 is located at the C-terminal end of the JM-B segment of the ERBB4 tyrosine kinase domain. In the ERBB4 asymmetric dimer tyrosine kinase domain structure [PDB code – 3BCE (40)], E715 of the receiver kinase forms part of the dimer interface and is mainly surrounded by polar residues from both the activator and receiver kinase domains (Fig. 5D). In the X-ray structure, residues near the E715 side chain in the receiver domain, include S789 (2.9 Å), Q793 (6.0 Å), and R720 (6.2 Å). Replacement of the negatively charged E715 to the positively charged lysine would also place E715K in the vicinity of S789 and Q793. However, with the aid of its long aliphatic side chain, lysine would be positioned to make an ionic interaction with E934 of the activator kinase domain, altering the interaction at the dimer interface (Fig. 5D). The E934–E715K interaction would consequently strengthen the activator-receiver kinase asymmetric binding, which would in turn help prolong the duration of the activated state of ERBB4 kinase. Because E934 is conserved among the ERBBs, E715K in ERBB4 could make similar ionic interactions when paired with EGFR, ERBB2, and ERBB3. However, EGFR and ERBB2 prefer to function as the receiver kinase when complexed with ERBB4 (63), in which case the E715K ERBB4 mutation may not have a significant impact. In contrast, in the ERBB3-ERBB4 heterodimer where ERBB3 serves as the activator kinase (63), E715K in ERBB4 would be positioned to interact with E925 in ERBB3, strengthening the heterodimer binding and RTK signaling.

To probe the dynamic nature of the E715K mutation on the ERBB4 homodimer structure, 100 ns MDS were carried out. Unlike simulation of the wild-type ERBB4 homodimer with E715, lysine of the E715K mutant in the receiver kinase is capable of forming an intermolecular hydrogen bond between the ε-amino side-chain group of E715K and the side-chain oxygen atoms of E934 in the activator kinase, observed during 31% of the simulation time. In the wild-type simulation, E715 of the receiver kinase mostly interacted with R720 (70%) and S789 (40%) within the receiver kinase. The energy contribution of residues E715 and E934 in the wild-type, and E715K and E934 in the mutant ERBB4—when considering all of the interactions the amino acids make with the rest of the residues in the dimer—shows that E715K (−1.97 ± 1.4 kcal/mol) and E934 (−0.92 ± 1.2 kcal/mol) of the mutant are more favorably placed than E715 (−0.82 ± 1.3 kcal/mol) and E934 (0.68 ± 1.2 kcal/mol) of the wild-type, as indicated by the lower average energy values of the residues in the mutant domains. Furthermore, analysis of the binding free energy between the ERBB4 monomers (Fig. 5E) showed an average of −67 ± 7.7 kcal/mol for the wild-type ERBB4 and −74 ± 8.4 kcal/mol for the E715K mutant, also suggesting stronger monomer–monomer interactions take place in the mutant ERBB4 homodimer. Collectively, the results show that the ERBB4 E715K mutation would strengthen the interactions in the dimer via formation of the K715–E934 ionic interaction, an effect that should increase the kinase activity.

To validate the *in silico* findings suggesting that ERBB4 E715K could serve as a more potent receiver kinase, we studied the mutant in an *in vitro* transactivation assay (Supplementary Fig. S10), with an approach similar to our previous study (14). Here, the ERBB4 E715K point mutation was introduced into an ERBB4 V954R mutant that is incapable of serving as the activator kinase, and thus cannot form a functional dimer with itself. For active signal transduction, the V954R mutant has to adopt the orientation of a receiver kinase in an asymmetric kinase dimer (Supplementary Fig. S10A and B; refs. 64, 65). As the structural analyses predicted that ERBB4 E715K could form stronger interactions at the dimerization interface with any of the ERBB family receptors, ERBB4 E715K was studied in the context of both ERBB4 homodimers as well as in heterodimers with other ERBB family members. To ensure the transactivation assay measures only the activity of the forced receiver kinase (ERBB4 V954R −/+ E715K), kinase-dead variants of EGFR (K721R; ref. 66), ERBB2 (K753M; ref. 67), and ERBB4 (K751R; ref. 68), as well as wild-type ERBB3 (intrinsically kinase-impaired), were introduced as activator kinases in the assay. In this system, performed in transiently transfected COS-7 cells, we were able to demonstrate that receiver kinase ERBB4, harboring the ERBB4 E715K mutation demonstrated greater activity regardless of the activator kinase ERBB (Fig. 5F; Supplementary Fig. S10C; *P* < 0.05), as indicated by the level of ERBB4 phosphorylation. ERBB4 homodimers promoted greatest ERBB4 activation, followed by EGFR, and ERBB3. Kinase-dead ERBB2, while producing an effect similar to that produced by other ERBBs as an activator kinase, was relatively inefficient in promoting ERBB4 phosphorylation (Fig. 5F; Supplementary Fig. S10C). It should be noted, that the transactivation assay cannot rule out the existence of potential oligomeric receptor complexes, which have been proposed in the context of ERBB3/ERBB2 heterodimers (69). These may also explain why the ERBB4 V954R-mutant receiver kinase is phosphorylated in the presence of kinase-dead ERBB partners, a situation resembling ERBB3/ERBB2 heterodimers. Together, the *in silico* and *in vitro* results demonstrate that ERBB4 E715K is a more efficient receiver kinase promoting increased transactivation in ERBB homodimers and heterodimers.

### R687K Mutation Can Stabilize Dimer Interactions at the Intracellular JM Segment of ERBB4

**FIGURE 6:**
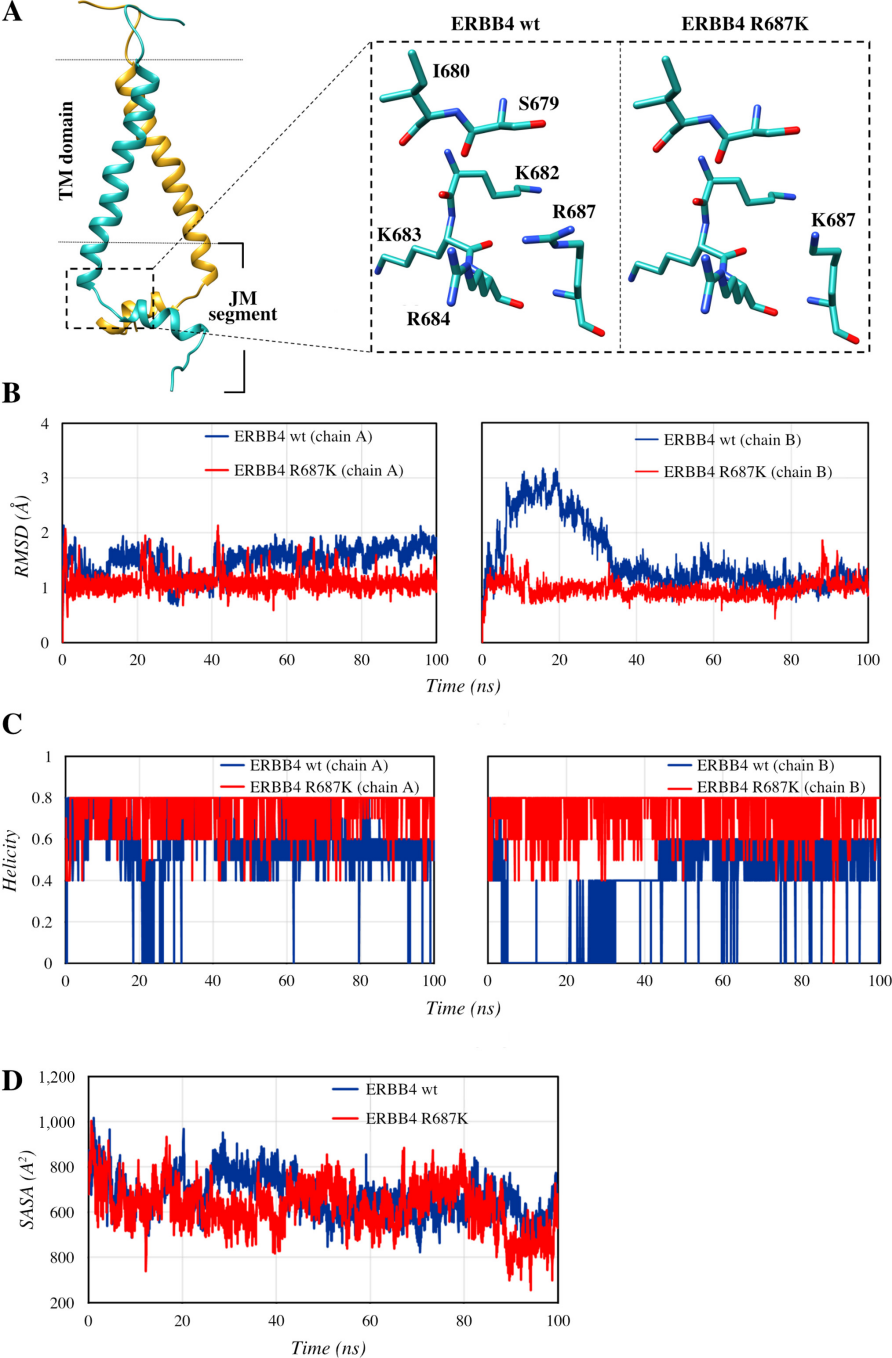
ERBB4 R687K. **A,** The ERBB4 TM-JM dimer structure (left) with the area near R687 enclosed in a small box. The residues surrounding R687 and K687 in the wild-type (wt) and mutant ERBB4 are displayed (right). **B,** Backbone atom RMSD of the JM C-terminal helix in ERBB4 dimer. **C,** The helical content of the JM C-terminal helix during the 100 ns simulations. **D,** The SASA for the interface residues of the antiparallel C-terminal helices during the simulation.

To unravel the mechanism of its enhanced activity, structural analyses were also carried out for ERBB4 R687K, the second most activating mutation based on the *in vitro* experiments. Arginine 687 is situated at the intracellular JM segment of ERBB4 (residues 676–713) that extends from the TM domain to the tyrosine kinase domain. The JM segment directly follows the TM domain and contains two helices: the N-terminal helix (676–681) and the C-terminal helix (685–694). These helices are separated by a linker loop (682–684), which is composed of three positively charged residues (Fig. 6A). Following the C-terminal helix is a loop, the JM latch, which constitutes a part of the tyrosine kinase core. In dimeric ERBBs, the C-terminal JM helix forms an antiparallel helix that plays a critical role in the dimerization and activation of ERBB kinases (70). In the ERBB4 TM-JM model structure (see the Materials and Methods for details), R687 is located at the JM C-terminal helix and the side-chain atoms are oriented toward the linker loop where hydrogen bonding interactions would be feasible with the main-chain oxygen atoms of S679, K682, and K683 (Fig. 6A). Replacement of R687 with lysine could still maintain these interactions with the backbone atoms (Fig. 6A); however, the ε-amino side-chain group of lysine is smaller in size unlike arginine's planar and bulky guanidinium group. Consequently, the mutant R687K could be more accessible to form frequent and additional interactions with the backbone oxygen atoms of S679 and I680 of the JM N-terminal helix. These interactions could in turn limit the movement of the C-terminal helix that is provided by the hinge loop. This positional stability of the JM C-terminal helix could play a key role in stabilizing the dimeric antiparallel JM helices, and as a result, promote ERBB4 activation.

The dynamic effects of the R687K mutation on the ERBB4 TM-JM dimeric structure were investigated by 100 ns MDS. During the simulations, R687 in wild-type ERBB4 mainly interacted with the side-chain oxygen atoms of E691 (47%), the main-chain oxygen atom of K682 (28.4%) and phosphate oxygen atoms of the membrane lipids POPS (89%) and POPC (23%) from the lower leaflet of the membrane bilayer. In contrast, R687K in the mutant ERBB4 is primarily hydrogen bonded with the main-chain oxygen atoms of S679 (23%) and I680 (33%) of the N-terminal helix. These two interactions may function as anchors connecting the N-terminal and C-terminal helices, imposing a positional restraint on the C-terminal helices. In the wild-type ERBB4 simulation, the bulkier guanidinium group of R687 did not form these interactions likely due to steric and geometric constraints, and hence R687 appears to be more suited for making interactions at the solvent-exposed surface.

The effects of the R687K mutation on the conformational dynamics of the ERBB4 antiparallel C-terminal helices were assessed by considering the backbone atom RMSDs relative to the initial structures. As shown in Fig. 6B, the mutant C-terminal helix of JM, chains A and B, exhibits higher stability (less structural variability) in comparison with wild-type ERBB4, with RMSDs of 1.09 ± 0.17 Å (chain A)/0.97 ± 0.15 Å (chain B), for the mutant, and RMSDs of 1.48 ± 0.26 Å (chain A)/1.53 ± 0.6 Å (chain B) for wild-type ERBB4. Furthermore, the alpha-helical content of the C-terminal helix was consistently higher for the mutant ERBB4 in comparison with the wild-type, suggesting that the helix in the R687K-mutant structure was stabilized to a higher degree than in the R687 wild-type structure (Fig. 6C). In line with this, the interfacial residues of the dimeric antiparallel helices were more tightly packed in R687K ERBB4, with an average solvent accessible surface area (SASA) of 622 ± 105 Å^2^ for the JM C-terminal helix, as compared with the wild-type ERBB4 with a higher average SASA of 669 ± 92 Å^2^ (Fig. 6D). The results from MDS collectively suggest that the ERBB4 R687K mutation stabilizes the JM helices and strengthens the antiparallel dimer packing via anchor-like hydrogen bonding interactions between R687K and residues of the N-terminal helix, which are lacking in the wild-type ERBB4. Consequently, the R687K mutation would be predicted to strengthen and hence prolong ERBB4 dimerization that is essential for tyrosine kinase activity.

## Discussion

With the development of over a hundred targeted cancer drugs (2), a critical clinical need has emerged for understanding the functional significance of individual somatic variants in oncogenes and tumor suppressor genes. This need has become increasingly evident with the information from large cancer genome sequencing efforts indicating that most statistically significant hotspot mutations are rare (22) and may co-operate with other functionally relatively weak somatic mutations (71). These observations suggest that statistical and structural data on the frequency, distribution, and localization of the individual mutants are not sufficient for making conclusion about their functional consequence.

Here we addressed the functional significance of 93.5% of all theoretically possible missense and nonsense mutations of a single cancer gene (72), *ERBB4*, in an unbiased functional genetics screen. The method exploiting error-prone PCR to generate random mutations for an expression library, and a model of ERBB4 activity-dependent cellular growth was based on the iSCREAM *(in vitro* screen for activating mutations) approach (29). The iSCREAM method was previously successfully used to analyze somatic evolution of random EGFR mutants during clonal expansion of the IL3-dependent Ba/F3 cells (29). ERBB4 provided a clinically relevant candidate for the effort, as it is a member of the well-characterized ERBB oncogene family but with an ambiguous role as a drug target by its own right (73–76), and as several recently approved pan-ERBB TKI drugs [afatinib, neratinib, dacomitinib (www.fda.gov), and poziotinib (77)] as well as other wide-spectrum RTK TKIs [such as ibrutinib (78, 79)] also target ERBB4. Moreover, hundreds of somatic mutations in *ERBB4* have been reported in different cancer types that are dispersed throughout the ERBB4 primary sequence in the absence of obvious mutation hotspots (Supplementary Fig. S1B). While the majority of these mutations may be passengers with low or no functional contribution to tumorigenesis, there is also accumulating evidence for existence for individual oncogenic ERBB4 variants (14, 15, 80).

Interestingly, some synthetic (these mutations have not been identified in cancer patients as of yet) constitutively activating ERBB4 mutations, such Q646C and I658E, have been demonstrated not to promote growth (81), or even promote apoptosis (82), *in vitro*. Taken together, these findings emphasize the importance of testing mutations simultaneously, using several models, and with unbiased technologies. The observations also underline the risk in trying to definitively categorize ERBB4 either as an oncogene or a tumor suppressor in the absence of information of both the biochemical nature of a particular ERBB4 variant and the context in which it is expressed. ERBB4 variants, such as E715K, that are particularly effective in transactivating heterodimeric ERBB partners, may for example stimulate differential intracellular signaling pathways and cellular outcomes, when co-expressed together within different ERBB heterodimers.

The screen identified 10 *ERBB4* mutations with enhanced activity in response to its ligand NRG-1. All the 10 mutations identified in the screen were at ERBB4 residues that have been reported to harbor mutations in patient-derived cancer samples listed in public cancer registries (COSMIC, cBioPortal, and AACR GENIE) with an identical missense mutation (same residue substituted with same residue) in 5 of the 10 (Supplementary Fig. S4).

The mutations in the 10 residues were found in cancers of 12 different tissues; most frequently in malignancies of the skin (*n* = 16), lung (*n* = 9), colon (*n* = 5), and head and neck (*n* = 3; Supplementary Fig. S4A; Supplementary Table S2). Although ERBB4 lacks obvious hotspots, the most frequent mutations are present in the ectodomain and in the intracellular JM domain (Supplementary Fig. S1B). ERBB4 mutations identified here also clustered in the intracellular JM domain and in the “C-terminal” tail (Supplementary Fig. S4B). These findings suggest that activating ERBB4 mutations are rare but present in clinical cancer samples.

All 10 ERBB4 mutants identified in our first round of ERBB4 iSCREAM were found to be more active than wild-type in NIH-3T3 upon serum starvation. Moreover, five of the 10 mutations were more potent in promoting ligand-dependent growth of Ba/F3 cells than wild-type ERBB4, and four of the 10 mutations demonstrated anchorage-independent growth advantage over wild-type ERBB4 in NIH-3T3 cells. In addition, four of the 10 mutations were rediscovered in a totally independent second round of the ERBB4 iSCREAM among the top 10 hits. Two of these four mutants (R687K and E715K) also promoted 3D growth of BEAS-2B cells and E715K promoted Ba/F3 allogenic tumor growth *in vivo*. These observations support the use of the iSCREAM method for identification of activating RTK mutations but also underline how different individual mutants with their particular signaling characteristics may promote differential cellular phenotypes depending on the context in which they are expressed.

To address the oncogenic signaling mechanisms of the individual variants, structural characteristics E715K and R687K were studied in detail. Interestingly, E715K that also demonstrated ligand-independent activity and robust potential to promote tumor formation *in vivo*, promoted enhanced kinase transactivation in ERBB4 homodimers and heterodimers, as suggested both by structural analyses of kinase dimerization interfaces as well as by an *in vitro* transactivation assay. The R687K mutation, on the other hand, was predicted to give positional stability for JM C-terminal helix, thereby stabilizing the antiparallel dimer packing of JM C-terminal helices of ERBB monomers. These findings are consistent with a model in which both of the mutations enhance interactions between receptor monomers that stabilize an active ERBB4 conformation.

In our recent work, we characterized a novel activating *ERBB2* mutation located in the kinase dimerization interface, the ERBB2 E936K (56). ERBB2 E936 is analogous to the E934 of ERBB4 and, based on the structural analysis, ERBB4 E934 in the activator kinase can make an ionic bond with the mutated E715K residue in the receiver kinase, thereby strengthening the ERBB4 asymmetric kinase dimer (Fig. 5D–F). Taken together, these findings suggest that glutamate-to-lysine mutations in the conserved residues at the asymmetric dimer interface of the ERBB kinase domains can effectively stabilize ERBB homodimers and heterodimers. It should be noted, however, that in this study, the role of ERBB4 E934 in the dimer interface was not directly experimentally addressed. Interestingly, the COSMIC, cBioPortal, and AACR GENIE cancer registries include several other examples of glutamate-to-lysine mutations at residues analogous to ERBB4 E715/E934 within the ERBB family genes. These include unique cancer patient samples harboring EGFR E709K (*n* = 67), EGFR E928K (*n* = 1), ERBB2 E717K (*n* = 10), ERBB3 E925K (*n* = 7), ERBB4 E715K (*n* = 8), and ERBB4 E934K (*n* = 5) variants. In accordance with the importance of this concept of induction of a stronger interaction within the kinase interface due to an ionic bond between glutamate and lysine residues, the EGFR E709K and ERBB2 E717K variants, analogous to ERBB4 E715K described here, have previously been characterized as oncogenic mutations (83–86).

While novel ERBB4 activating mutations were discovered, the screen also failed to identify previously reported activating ERBB4 mutations, such as K935I (14). This may be due to the known tissue-specificity in the activity of oncogenes (87). While optimal for screening kinase-dependent growth (55), it is important to keep in mind that the Ba/F3 cells used here as the cellular background for the screen are murine hematopoietic cells (54), and thus considered ontologically distant from lineages that form solid tumors. A factor that could affect the selection pressure to favor specific ERBB4 mutations is for example the fact that Ba/F3 cells endogenously express a small amount of ERBB3 (29, 57), another NRG receptor and a heterodimeric partner for ERBB4. Another plausible explanation could be that strong activating mutations outcompete the weaker variants during the evolution of fastest-growing cell clones, similar to what was suggested in our earlier iSCREAM analysis of EGFR mutants that demonstrated a disproportionate variant frequency for the major oncogenic EGFR variant L858R (29). As an outcome of strong mutations dominating weaker ones, one could potentially also miss functionally moderate mutations that, to obtain full activity, require another co-operating mutation *in cis* within the same allele (71) or in the heterodimerizing partner ERBB. Interestingly, a recent report by Saito and colleagues indicated that ERBB encoding genes frequently harbor multiple driver mutations *in cis*. In particular, several hotspot mutations of EGFR which are implicated in non–small cell lung cancer, were discovered to co-occur *in cis* with other EGFR mutations (71). While the mutation frequency was 2.59 nucleotide changes per ERBB4 insert in our iSCREAM analysis, the read length in the deep sequencing of the data (30–151 bp) did not allow for addressing the putative co-operative function of multiple *ERBB4* mutations in the *ERBB4* JM-a CYT-2 insert (3,879 bp). The significance of multiple *ERBB4* mutations and the eventual frequency of functional ERBB4 mutations in the different cancer sample series thus remain to be elucidated.

It is of note that the mutation frequency of 2.59 nucleotide changes per ERBB4 insert (translating to an average of 1.79 amino acid residue changes per ERBB4 polypeptide) also carries the risk of co-occurring mutations of which only one contributes to cell survival, while the other(s) serve as pure passenger mutation(s). Other factors carrying the risk of false-positive findings include the variation in expression levels between individual variants (29), as well as putative integration of the inserts to genomic loci with functional relevance (88). These phenomena are also plausible explanations for the observed differences in the two independent rounds of the ERBB4 iSCREAM. To be able to address the presence of putatively co-operating or nonfunctional co-existing mutations in the cDNA inserts promoting the clonal expansion, future development of the iSCREAM approach should include leveraging modern deep sequencing technologies capable of analyzing reads up to the length of the whole cDNA of interest.

Taken together, our functional screen identified novel activating ERBB4 mutations that are sensitive to clinically approved pan-ERBB TKIs. Together with our previous screen on EGFR, the data further indicate that the iSCREAM pipeline can be used to identify rare activating mutations in other oncogenes in an unbiased manner not influenced by the mutation frequency.

## Supplementary Material

Table S1 Excel sheet listing all the constructs generated to express indicated ERBB mutants.

Table S2 Excel sheet showing the details of the mutations represented in Figure S4.

Supplementary Data Supplementary Figures and Supplementary Methods
