# Supplementary material for: An Unbiased Functional Genetics Screen Identifies Rare Activating ERBB4 Mutations
Source: Cancer Res Commun. 2022 Jan 7;2(1):10–27. doi: 10.1158/2767-9764.CRC-21-0021 (PMC9973412; doi:10.1158/2767-9764.CRC-21-0021)
Supplement: Supplementary Data — Supplementary Figures and Supplementary Methods [file crc-21-0021-s03.pdf]

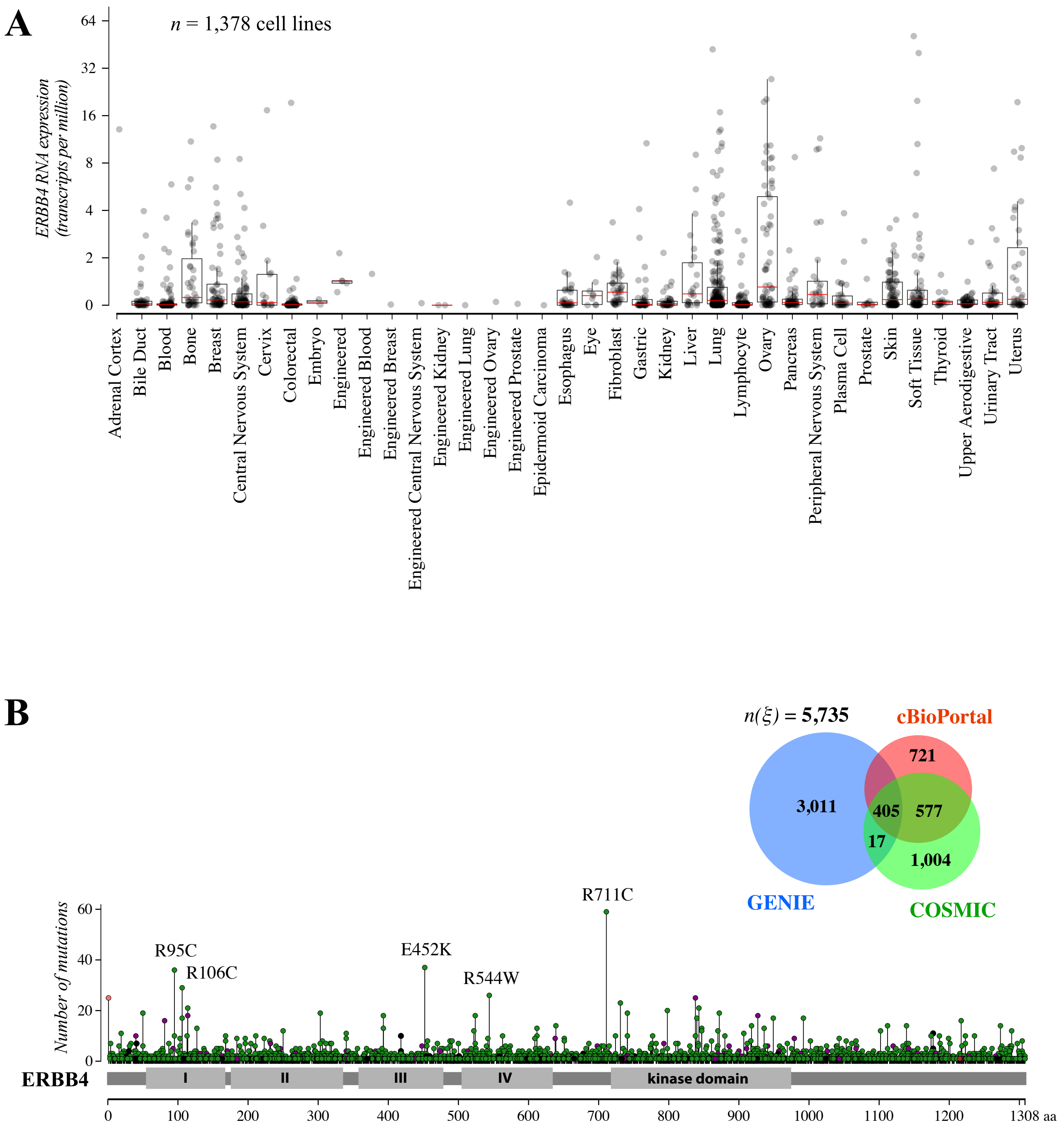

Supplementary Figure S1. *ERBB4* expression and mutations in cancer. A) Expression of *ERBB4* transcripts in 1,378 cancer cell lines according to RNAseq data analyzed in the DepMap project (https://depmap.org). Please note that the *y*-axis ticks are placed on a log2 scale. B) Unique ERBB4 coding mutations (missense, nonsense, frameshift, small insertions and deletions) reported in COSMIC (https://cancer.sanger.ac.uk), AACR GENIE (https://genie.cbioportal.org), and cBioPortal databases (https://cbioportal.org) covering various cancer histologies. The *x*-axis shows the position of different residues on the ERBB4 primary sequence. Five most frequent mutations are labelled. The Venn diagram shows the distribution of the 5,735 mutations among the databases.

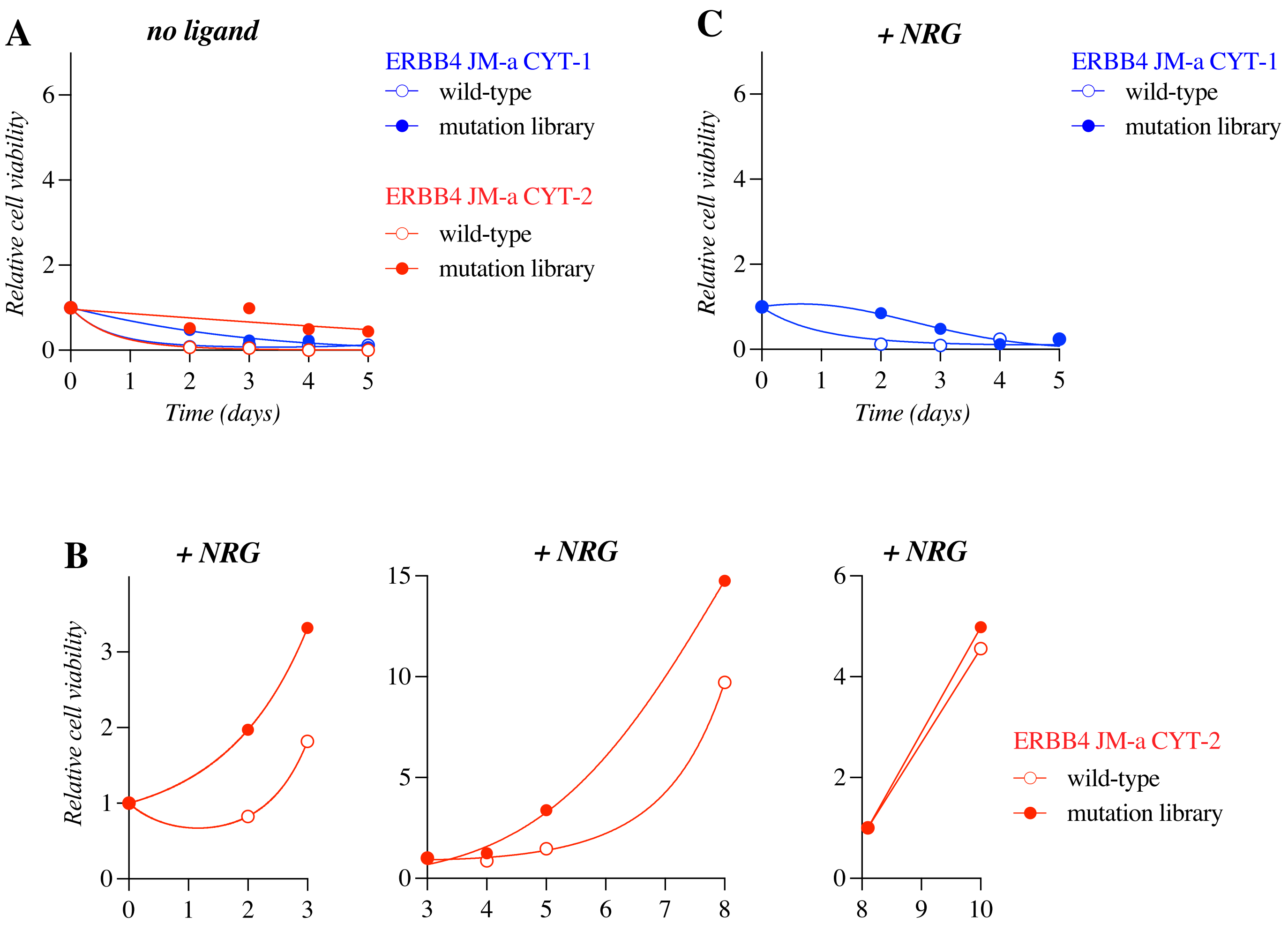

Supplementary Figure S2. Random mutations in ERBB4 JM-a CYT-1 *vs* JM-a CYT-2 isoforms. Ba/F3 cells expressing wild-type ERBB4 JM-a CYT-1 or ERBB4 JM-a CYT-2, or their respective randomly mutated expression libraries, were cultured in the presence or absence of 10 ng/ml NRG-1 in the absence of IL-3. Viable cells were counted with trypan blue and TC20 automated cell counter (Bio-Rad). Relative viability refers to the cell count at the indicated time point divided by the cell count at the beginning. The three panels in B indicate three successive passages of the cells. An equal number of cells were seeded on days 0 (left panel), day 3 (middle panel), and on day 8 (right panel).

A Mutation distribution at nucleotide level

Rows: mutation library; columns: wild-type *ERBB4*

mutation library \ wild-type *ERBB4*	A	C	G	T
A	A	750	477	763
C	689	C	464	773
G	478	422	G	723
T	579	612	666	T

number of mutations

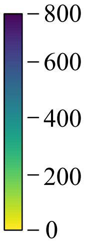

B Mutation distribution at amino acid level

Rows: ERBB4 mutation library; columns: wild-type ERBB4

ERBB4 mutation library \ wild-type ERBB4	*	A	C	D	E	F	G	H	I	K	L	M	N	P	Q	R	S	T	V	W	Y
*	*		53		77		35			58	30				44	30	13			15	50
A		A		66	77		64							74			39	68	76		
C			C			47	34									14	56			16	50
D		38		D	80		35	25					74						34		49
E	0	24		66	E		43			61					36				39		
F			52			F			54		61						27		32		50
G		55	51	66	78		G									57	35		75	15	
H				52				H			32		72	47	42	14					50
I						46			I	32	49	26	72			20	35	61	42		
K	0				78				12	K		26	72		44	27		25			
L	0					47		28	66		L	26		84	42	36	12		66	15	
M									63	28	59	M				7		5	32		
N				67				30	54	59			N				34	42			43
P		54						28			77			P	45	31	39	69			
Q	0				65			29		57	46			37	Q	24					
R			52				72	29	12	61	77	26		72	45	R	35	16		15	
S	0	62	50			47	33		53		30		74	82		40	S	69		12	50
T		62							67	62		26	73	84		23	60	T			
V		62		66	78	44	78		67		90	26							V		
W			45				9				19					22	2			W	
Y	0		52	67		47		30					74				27				Y

number of mutations

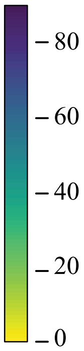

* STOP codon

☐ not possible by changing single nucleotide

Supplementary Figure S3. Distribution of mutations in the expression library. A) Distribution of observed nucleotide changes in the ERBB4 mutation library. B) Distribution of observed amino acid changes in the ERBB4 mutation library. In both matrices, the cells at the intersection of the labels on the columns and the rows show the number of mutations with that specific change (nucleotide or amino acid).

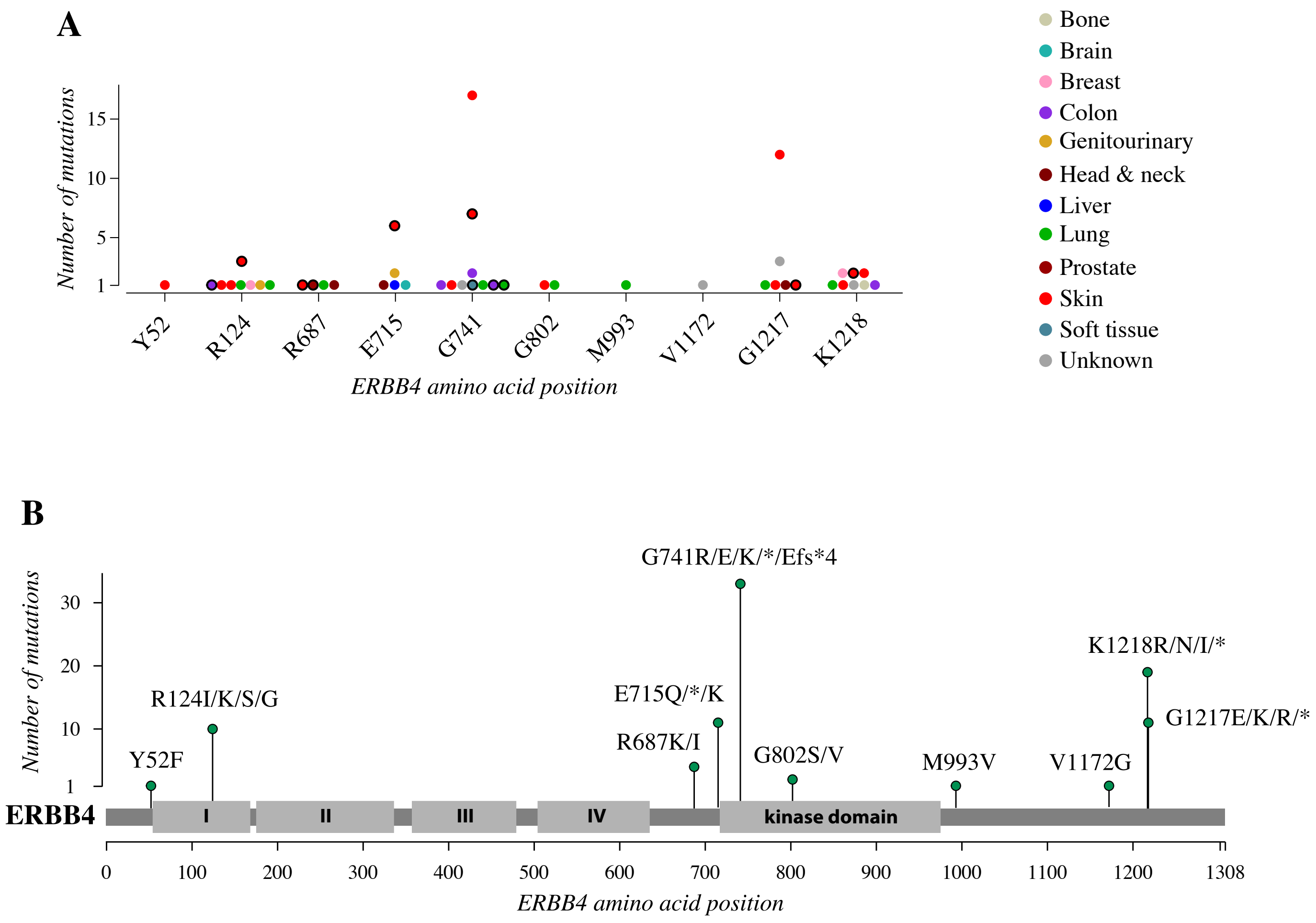

Supplementary Figure S4. Frequency of ERBB4 mutations identified in the *in vitro* screen in clinical cancer samples. ERBB4 mutations found in clinical cancer samples and listed in the three databases AACR GENIE, cBioPortal and COSMIC are shown (overlapping data removed). A) Tissue-specific frequency of unique mutations at the indicated ERBB4 residues in the three databases represented as a dot plot. The color of the dot represents the tissue of origin for the human cancers and a solid border of the dot indicates that the amino acid change was exactly the same as identified in our screen (Figure 1). B) Cumulative frequency of mutations in any cancer tissue at the indicated ERBB4 residues in the three cancer databases represented as a lollipop diagram on the ERBB4 primary sequence.

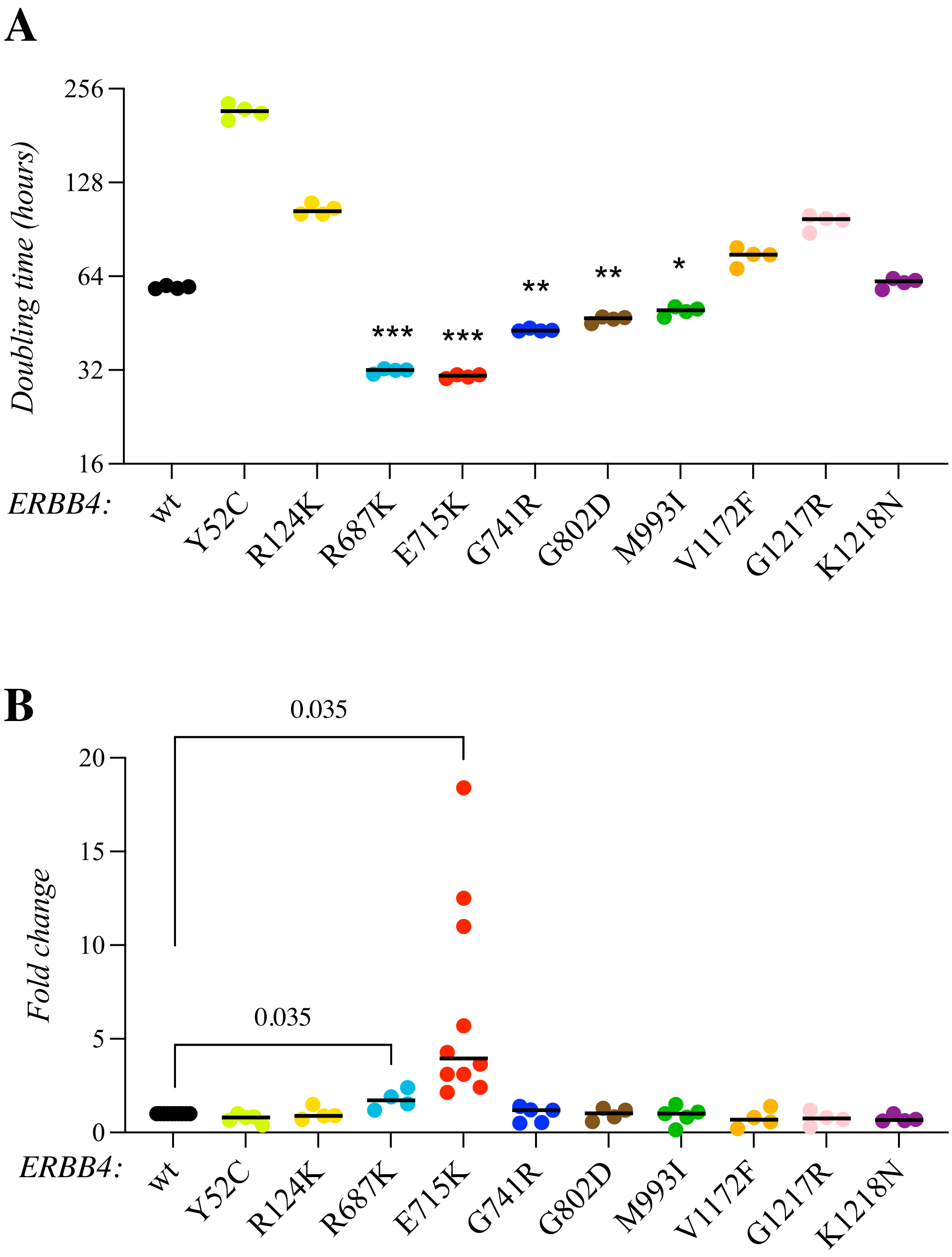

Supplementary Figure S5. Quantification of activity of ERBB4 variants expressed in Ba/F3 cells. A) Effect of ERBB4 variants on Ba/F3 cell doubling time. Ba/F3 cells expressing the indicated ERBB4 variants were cultured in the presence of NRG-1. Cell viability was analyzed with the MTT assay in quadruplicates. Welch Two Sample t-test was used to calculate whether the doubling time was statistically significantly reduced as compared to cells expressing wild-type ERBB4. *, $q < 10^{-3}$; **, $q < 10^{-5}$; ***, $q < 10^{-7}$. B) Densitometric quantification of ERBB4 phosphorylation relative to ERBB4 expression in western analyses of Ba/F3 cells expressing the indicated ERBB4 variants. Data are from different experiments testing basal ERBB4 phosphorylation in the absence of NRG-1 stimulation.

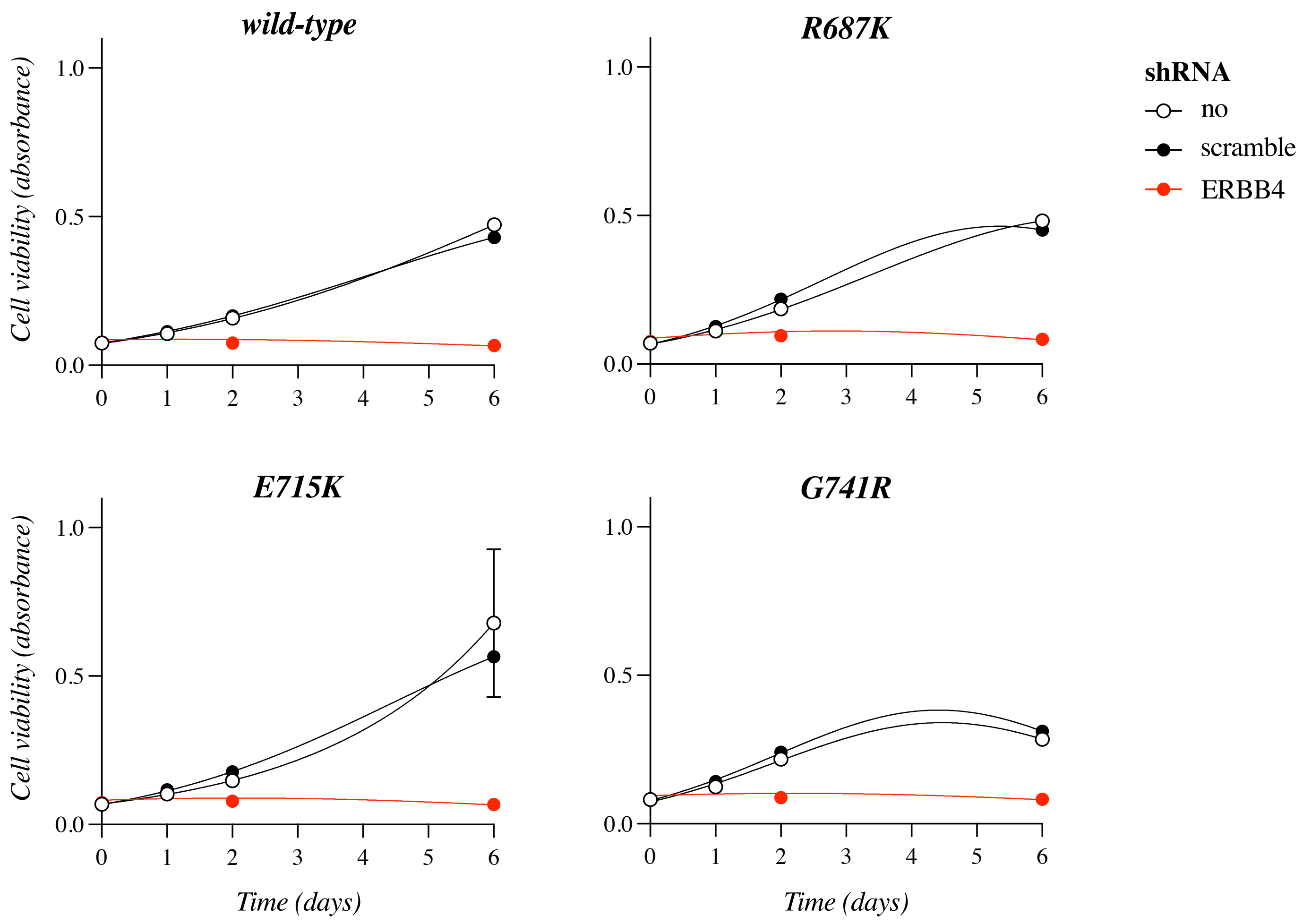

Supplementary Figure S6. Validation of ERBB4-dependency of the NRG-dependent Ba/F3 clones expressing ERBB4 variants. Ba/F3 cells expressing wild-type ERBB4 or the indicated ERBB4 variants were transduced with an ERBB4-targeted shRNA, scrambled shRNA, or incubated with Polybrene only (no shRNA). Cells were cultured in the presence of NRG-1. Cell viability was analyzed with the MTT assay. The mean and standard deviation of quadruplicate analyses are shown.

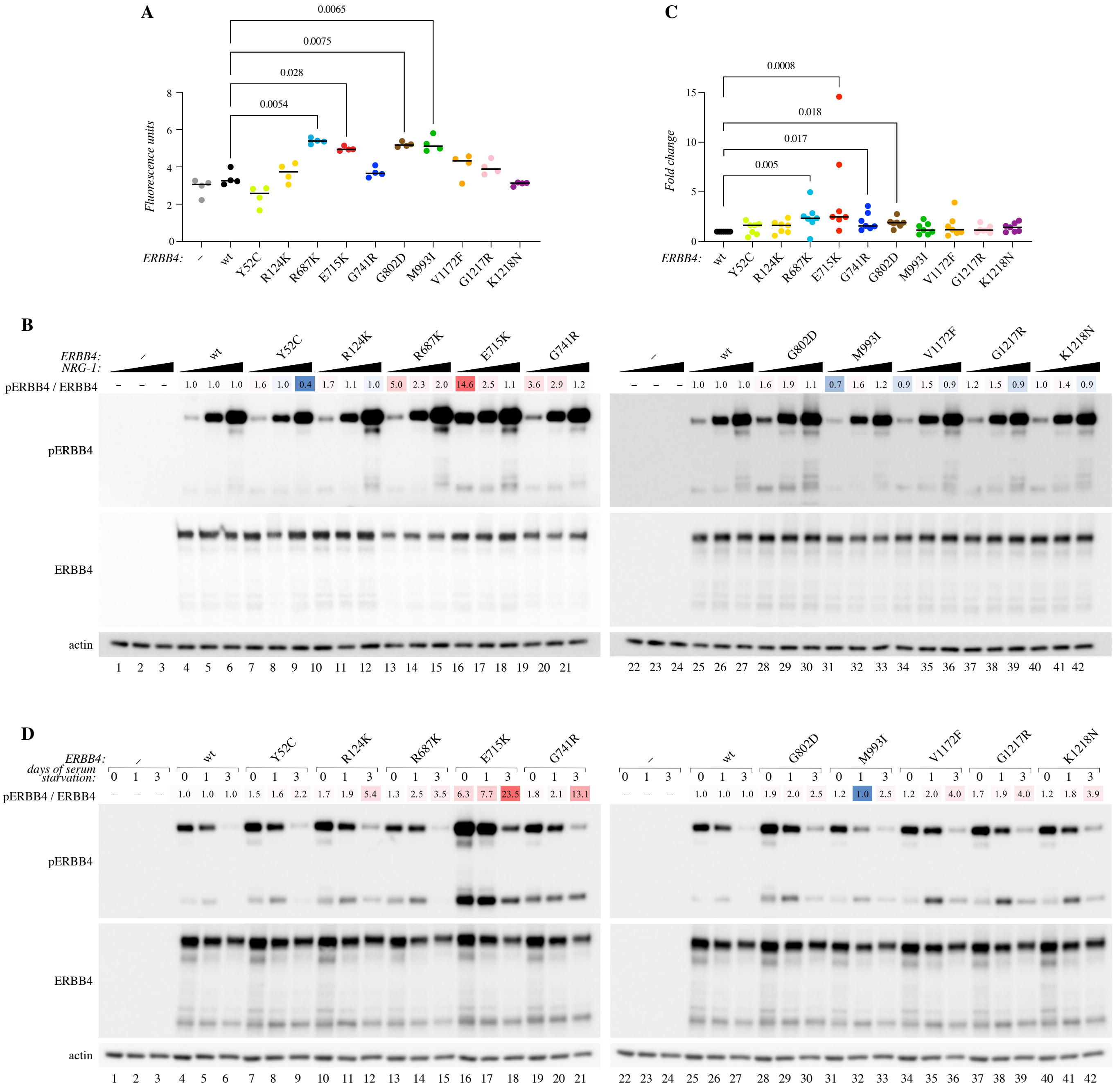

Supplementary Figure S7. Validation of the screen in NIH-3T3 cells. A) NIH-3T3 cells stably expressing wild-type (wt) ERBB4 or the indicated ERBB4 variants, or vector control cells (-) were analyzed for anchorage-independent growth in soft agar in the presence of 10% FCS and 50 ng/ml NRG-1. Cell viability was analyzed using alamarBlue after culturing the cells for 11 days. Data of quadruplicate analyses are presented as a dot plot, with horizontal lines indicating the median. Statistically significant *P* values (*P* < 0.05) for pairwise comparisons between the wild-type and the other cell lines are shown (Brown-Forsythe and Welch ANOVA tests, Dunnett's T3 multiple comparisons test). B) NIH-3T3 cells stably expressing the indicated constructs were starved in 0% FCS overnight, and treated with 0, 10 or 50 ng/ml NRG-1 for ten minutes and analyzed for ERBB4 phosphorylation by western blotting. Heatmap indicates fold changes in ERBB4 phosphorylation normalized to ERBB4 expression. C) Densitometric quantification of ERBB4 phosphorylation relative to ERBB4 expression in western analyses of NIH-3T3 cells expressing the indicated ERBB4 variants. Data are from different experiments testing the effects of NRG-1 stimulation and serum-starvation on ERBB4 phosphorylation. Statistical analyses were carried out using the Kruskal-Wallis test. To correct for multiple testing, the false discovery rate was controlled using two-stage step up method of Benjamini, Krieger and Yekutieli. Statistically significant q values (q < 0.05) are shown. D) NIH-3T3 cells expressing the indicated constructs were starved in 0% FCS for 0, 1 or 3 days, and analyzed by western blotting as in B. Heatmap indicates fold changes in ERBB4 phosphorylation normalized to ERBB4 expression.

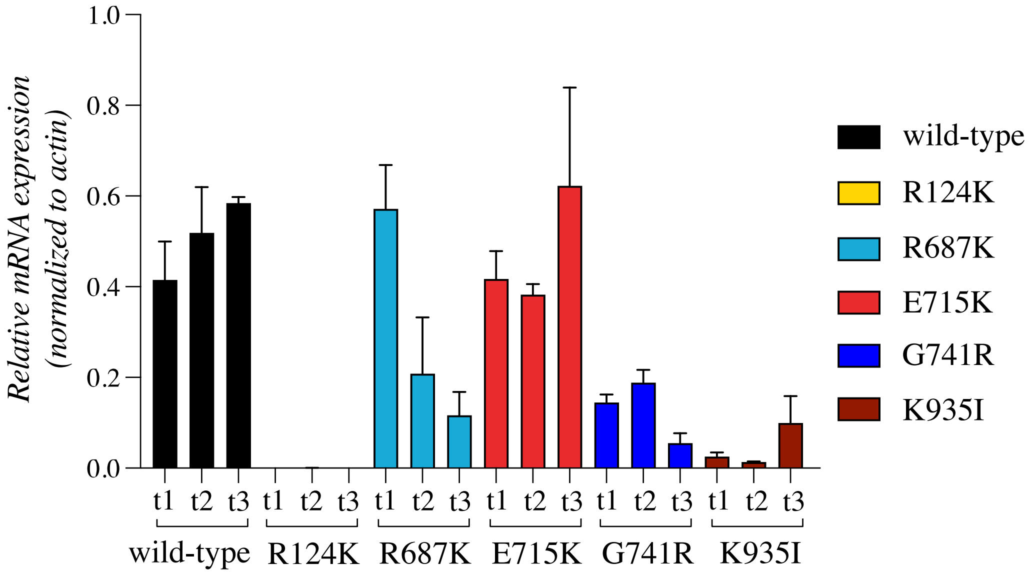

Supplementary Figure S8. *ERBB4* mRNA expression in allogenic Ba/F3 tumors. A) Tumor tissues from sacrificed mice were analyzed for *ERBB4* mRNA expression using real-time RT-PCR. Mean and standard deviation of duplicate samples from three tumors per ERBB4 variant are shown. Data are expressed relative to mouse β-actin mRNA expression as a reference.

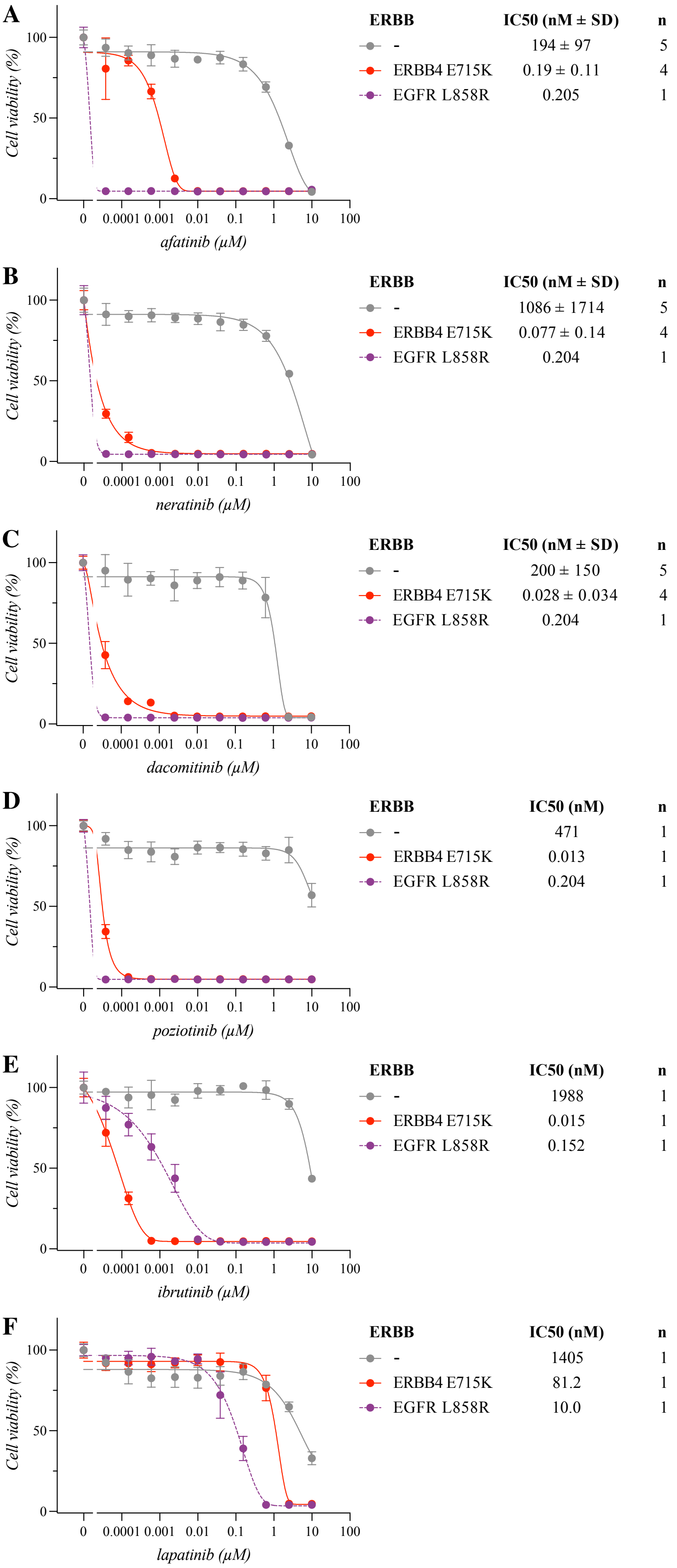

Supplementary Figure S9. Sensitivity of ligand-independent Ba/F3 cells expressing ERBB4 E715K to tyrosine kinase inhibitors. Ba/F3 cells expressing the ERBB4 E715K or the known oncogenic EGFR L858R were cultured for three days in the presence of the indicated concentrations of ERBB TKIs. Cells expressing ERBB4 E715K or EGFR L858R were cultured in the absence of both NRG-1 and IL-3. The vector control cells were cultured in the presence of IL-3. Cell viability was measured with MTT assays. The mean and standard deviation of quadruplicate analyses are shown for the dose response curves. IC50 values for the drug responses were calculated from the indicated number of independent analyses (n) after fitting the dose-response curves with four-parameter log-logistic function (R; 'drc' package).

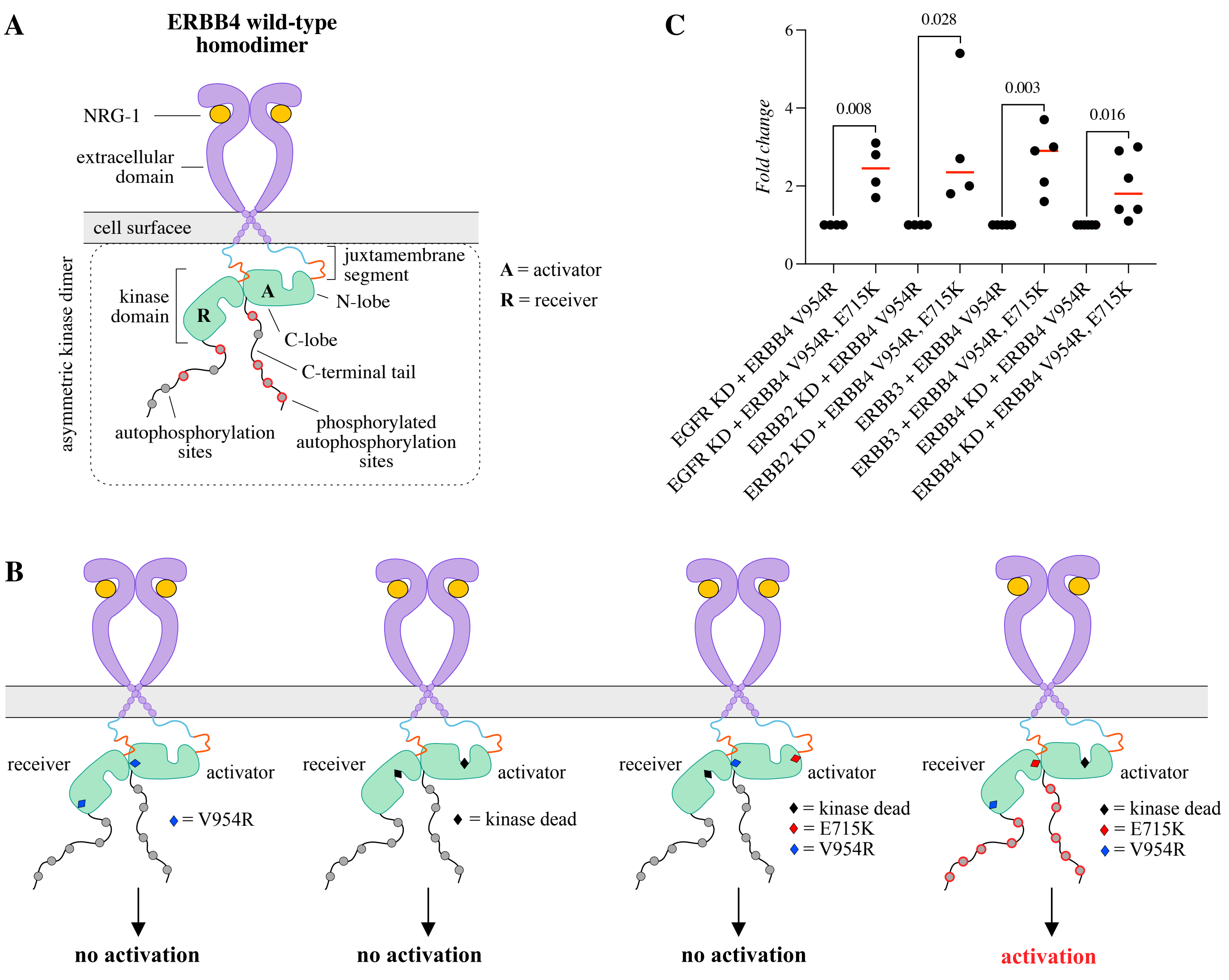

Supplementary Figure S10. Transactivation by ERBB4 E715K receiver kinase activity. A) A schematic diagram showing an active NRG-1-bound ERBB4 homodimer with an asymmetric kinase dimer formed between the intracellular domains. B) E715 is localized in the N-lobe of the receiver kinase at the kinase dimer interface. To address the transactivation activity of the ERBB4 E715K mutant, its ability vs. wild-type ERBB4 to promote ERBB4 autophosphorylation was studied *in cis* with a V945R mutation known to block the ability of the kinase to function as the activator. To include a potent activator kinase in the assay, the E715K/V945R double mutant was co-expressed *in trans* with the kinase-dead K751R ERBB4, which in turn is incapable of functioning as the receiver kinase. Thus, in the experimental set-up, neither of the constructs can promote autophosphorylation when expressed alone, while the combination shown on the right can dissect out receiver kinase-specific transactivation activity of a mutant, such as E715K, introduced into the V945R context. Positions of the various mutations are shown with diamond shapes. Mutations abrogating kinase activation have the autophosphorylation sites in the C-terminal tail colored grey, while phosphorylated autophosphorylation sites are indicated as grey dots circled with red borders. C) Densitometric quantification of ERBB4 phosphorylation relative to ERBB4 expression in transactivation assay western blots. Data are presented as ERBB4 E715K/V954R double mutant value fold change to that of the V954R mutant. Pairwise comparisons were made separately for each co-transfected ERBB by one-sample t-test from log-transformed data. Statistically significant P values ($P < 0.05$) are shown.

Supplementary Methods

Lentiviral-mediated ERBB4 knockdown of NRG-dependent stable Ba/F3 cell lines

To knock down ERBB4 expression in NRG-dependent Ba/F3 cell lines stably expressing wild-type ERBB4 or indicated ERBB4 mutants were lentivirally transduced as described previously (1), with the exception of using 0.8 μg/ml Polybrene. The following shRNA constructs were used: scrambled shRNA (a gift from David Sabatini; Addgene plasmid #1864; http://n2t.net/addgene:1864; RRID:Addgene_1864 (2)) or ERBB4-targeting shRNA (TRCN0000001411, Sigma-Aldrich). Mock transduction was carried out with 0.8 μg/ml Polybrene only.

Real-time RT-PCR

Total RNA was extracted from tumor tissues with NucleoSpin TriPrep kit (Macherey-Nagel) according to the manufacturer's protocol. Real-time RT-PCR analysis was carried out as described previously (3), except that the following primers and probes were used: human ERBB4 CYT forward 5'-caacatcccacctcccatctatac-3' (Pharmacia), human ERBB4 CYT reverse 5'-acactccttgttcagcagcaaa-3' (Pharmacia), human ERBB4 CYT-2 probe 5'-FAM-aattgactcgaataggaaccagtttgtataccgagat-TAMRA-3' (Eurogentec), mouse β-actin forward 5'-ctaaggccaaccgtgaaaag-3' (Eurofins Genomics), mouse β-actin reverse 5'-accagaggcatacagggaca-3' (Eurofins Genomics), and Universal ProbeLibrary probe #64 (Roche). Samples were analyzed in duplicates and *ERBB4* mRNA expression relative to the expression of the mouse β-actin reference gene was determined.

References (Supplementary Methods):

1. Ojala VK, Knittle AM, Kirjalainen P, Merilahti JAM, Kortesoja M, Tvorogov D, et al. The guanine nucleotide exchange factor VAV3 participates in ERBB4-mediated cancer cell migration. J Biol Chem. 2020;295:11559–71.

2. Sarbassov DD, Guertin DA, Ali SM, Sabatini DM. Phosphorylation and regulation of Akt/PKB by the rictor-mTOR complex. Science. 2005;307:1098–101.

3. Chakroborty D, Kurppa KJ, Paatero I, Ojala VK, Koivu M, Tamirat MZ, et al. An unbiased in vitro screen for activating epidermal growth factor receptor mutations. J Biol Chem. 2019;294:9377–89.
